# Proliferation and differentiation in intestinal organoids are balanced by ligand-modulated EGFR trafficking

**DOI:** 10.26508/lsa.202603733

**Published:** 2026-08-27

**Authors:** Mario O Caracci, Sabrina Seidler, Luis M Muñoz-Nava, Birga Soetje, Kirsten Michel, Philippe IH Bastiaens

**Affiliations:** Department of Systemic Cell Biology, Max Planck Institute for Molecular Physiology , Dortmund, Germany

## Abstract

Epidermal growth factor (EGF) signaling is associated with proliferation and tumorigenesis. Conversely, EGF-family ligands can also trigger a differentiation program, an effect attributed to ligand affinity and EGF receptor (EGFR) activity. Most of these observations have been made in immortalized cell culture experiments, whereas the mechanisms underlying EGF/EGFR-driven proliferation-differentiation dynamics in complex development and tissue self-renewal have not been addressed. We show that culturing mouse small intestinal organoids (mSIOs) without EGF enhanced EGFR expression and basal phosphorylation while maintaining a balanced development of proliferative crypts and differentiated villi. Addition of EGF or Epiregulin (EREG) triggered receptor endocytosis, reducing cell-surface levels and expression. While EGF promoted crypt proliferation, EREG promoted both proliferation and villus differentiation compared with untreated controls. Removal or re-introduction of EGF or EREG proved sufficient to induce development comparable to the constant presence of ligands over 96 h. Sub-saturating concentrations of EGF led to increased villus differentiation, resembling EREG treatments, suggesting that control over EGFR endocytic cycle regulates its plasma membrane localization shaping the balance of proliferation and differentiation in mSIOs.

## Abstract (precis)

Medium ligand-composition and concentration modulates EGFR signaling competency at the plasma membrane, balancing crypt proliferation and villus differentiation, enhancing mouse intestinal organoids self-renewal.

## Introduction

Epidermal growth factor (EGF)/EGF receptor (EGFR) signaling is essential for proliferation and self-renewal of intestinal stem cells (ISCs). Aberrant activity of EGF downstream signaling has been consistently associated with oncogenic transformations (Tomas et al, 2014). EGF is a fundamental growth factor for many in vitro organoid culture systems (Mahe et al, 2013; Hu et al, 2018; van der Vaart et al, 2021; Wang et al, 2025). In mouse small intestinal organoids (mSIOs), Paneth cells secrete paracrine EGF within the stem cell niche at the crypt base (Sato et al, 2011a). Nevertheless, additional growth factors, including EGF, are added to the culture medium, mimicking the complexity of the stromal compartment surrounding the intestinal epithelia (Sato et al, 2009).

In the intestine, EGFR is mainly expressed in the crypts (Yang et al, 2017) and inhibition of tyrosine kinase activity in mature organoids led to cell cycle arrest and quiescence of LGR5+ stem cells (Basak et al, 2017). EGFR inhibition also enhanced differentiation toward enteroendocrine cells, suggesting that perturbations in EGF/EGFR activation during distinct developmental stages could alter differentiation in mSIOs (Basak et al, 2017). Other EGF-family ligands such as Neuregulin (NRG1), Epiregulin (EREG) and Heparin-binding EGF-like growth factor (HB-EGF) can replace EGF in mSIOs culture, promoting organoid development (Chen et al, 2012; Jardé et al, 2020; Lemmetyinen et al, 2023), notably epithelial knock-out of NRG1 does not affect differentiation of secretory lineage cells (Jardé et al, 2020). While NRG1 does not bind EGFR, EREG binds both EGFR and receptor tyrosine-protein kinase erbB-4 (ERBB4) and has been used in human intestinal organoids to enhance cellular differentiation and crypt development (Childs et al, 2023).

Under basal medium conditions, where the medium contains EGF, Noggin, and R-spondin, mSIOs show both crypt proliferation and villus differentiation in two distinct, spatially separated compartments (Sato et al, 2009). In contrast, human intestinal organoids feature less cellular and structural diversity. Several remarkable efforts have been taken to enhance human organoid complexity by modulating cell signaling, including EGF inhibitors and EGF-family ligands (Sato et al, 2011b; Fujii et al, 2018; He et al, 2022; Yang et al, 2025). Nevertheless, the spatiotemporal responses of dynamic intracellular signaling networks to extracellular ligands have been largely overlooked.

Located at the plasma membrane (PM), EGFR is the main signaling hub decoding the inputs from EGF-family ligands. It is well established that EGFR signaling plays an integral role in partially opposing phenotypic outcomes, including migration, apoptosis, proliferation, and differentiation, and it is generally acknowledged that these responses are a result of different affinities among EGF-family ligands and their capacity to induce receptor phosphorylation (Abud et al, 2021; Jendrisek et al, 2026). For instance, high-affinity ligands such as EGF, HB-EGF, and transforming growth factor alpha (TGFα), all produced in the intestinal epithelia, are often associated with cell proliferation (Abud et al, 2021; Jendrisek et al, 2026). On the other hand, low-affinity ligands such as EREG and Amphirgulin (AREG), which are expressed by stromal cells in the intestine, are associated with cell differentiation and migration (Lemmetyinen et al, 2023; Jendrisek et al, 2026). Interestingly, EGF-triggered responses are dependent on ligand concentration, where high, receptor-saturating concentrations promote cellular proliferation while low, sub-saturating concentrations promote migratory behavior in cultured cells (Brüggemann et al, 2021; Joshi et al, 2023).

The fact that EGF-EGFR signaling can trigger multiple phenotypic responses underscores the importance of this ligand-receptor pair during development. However, these phenotypic responses have mainly been demonstrated in immortalized cell lines, lacking the decision-making dynamic of complex developing tissues under homeorhetic conditions. While animal knock-out models have certainly shown that EGF-family ligands and EGFR are important for development (Abud et al, 2021), they do not allow for the systematic interrogation of ligand abundance or EGFR membrane/endosomal localization in the context of ongoing development.

Therefore, we studied mSIOs in early developmental stages during self-renewal and regeneration from seeded single crypts and their subsequent maturation. For this, we cultured mSIOs with either EGF or EREG to examine the differential effects of ligand availability on the maintenance of organoid crypt and villus development. Our findings PM EGFR localization drives villus development regardless of ligand affinity, suggesting a preponderant role for EGFR endocytic cycle in intestinal development.

## Results

### EGF promotes crypt proliferation and EREG promotes villus differentiation

**Figure 1.**
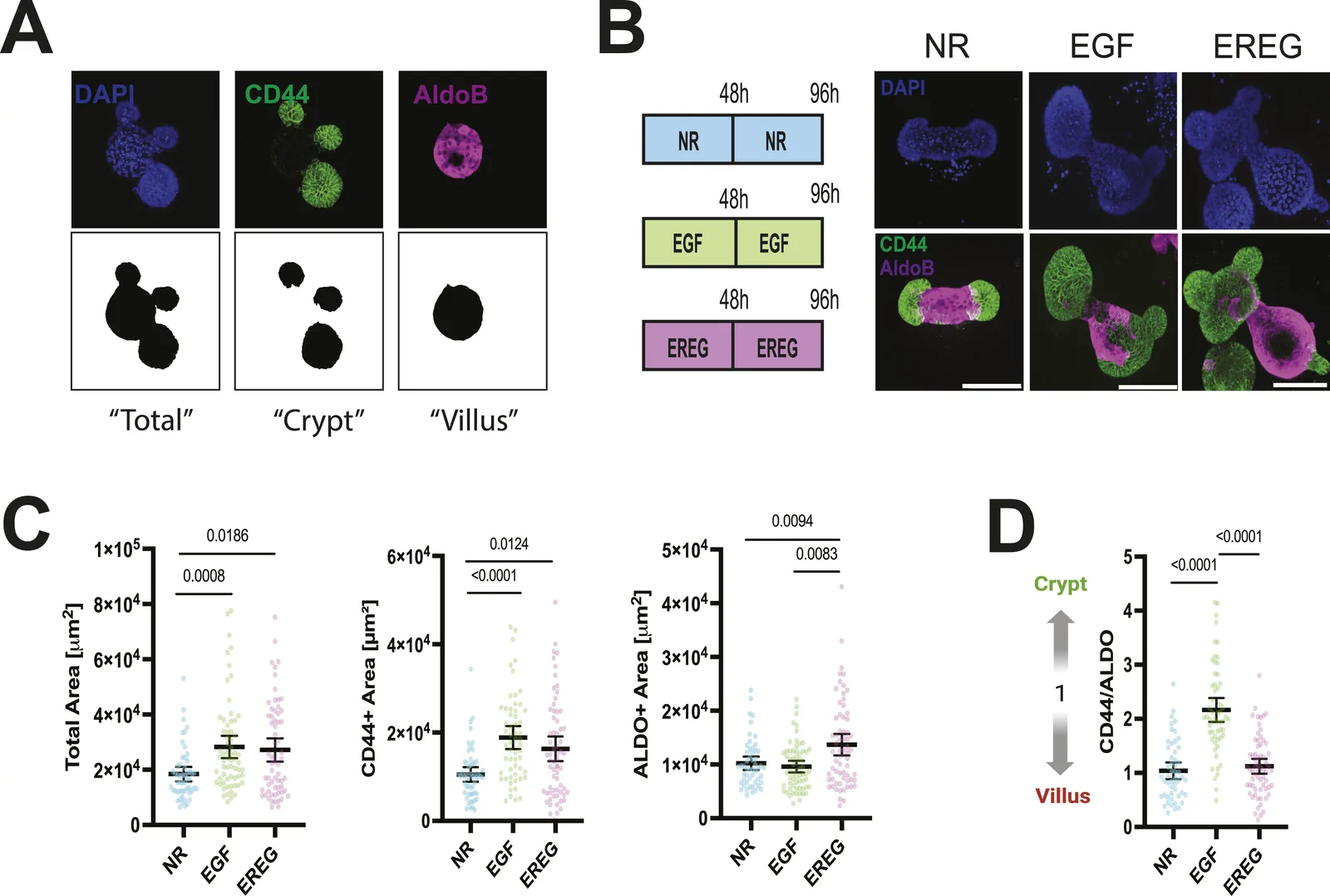
EGFR surface depletion by EGF promotes crypt proliferation while EREG promotes both proliferation and differentiation. **(A)** Maximum intensity projections (Top) were binarized (Bottom) to quantify the area of DAPI (nuclei, Blue), CD44 (Green), and AldoB (Magenta) used to quantify “Total,” “Crypt” and “Villus” area respectively. **(B)** Organoids were grown, with NR medium or supplemented with either EGF (50 ng/ml) or EREG (50 ng/ml) for 96 h with a complete medium change at 48 h. Organoids were immuno-stained with CD44 (Green), AldoB (Magenta), and DAPI (Blue). Scale Bar 100 μm. **(C)** Quantification of the “Total,” “Crypt” and “Villus” areas as shown in (A). Scatter plots with mean ± 95% CI. N = 3; n = 57–67 organoids/condition; significance was calculated by Kruskal-Wallis with Dunn’s multiple comparisons test. *P*-values <0.05 are shown. **(D)** Relative ratio of CD44+ to ALDO+ areas from Scatter plot with mean ± 95% CI; significance was calculated by Kruskal-Wallis with Dunn’s multiple comparisons test. *P*-values <0.05 are shown. Values above 1 suggest organoid development tends toward crypt proliferation, while values below 1 tend to villus differentiation.

To understand how the balance between crypt proliferation and villus differentiation is triggered by distinct EGF-family growth factors, we stained organoids with CD44, a bona fide crypt marker (Sumigray et al, 2018), and enterocyte marker AldoB to identify the differentiated villus region (Serra et al, 2019). While the villus region holds several differentiated cell types that arise from cycling cells located in the crypt, we chose AldoB+ enterocytes to measure organoid differentiation, as enterocytes are the most abundant cell type in the intestine, comprising about 70% of cells in the human ileum (Wang et al, 2019; Cosovanu et al, 2022). Images were acquired by Confocal Laser Scanning Microscopy (CLSM), and maximum-intensity projections from multiple confocal stacks were masked and quantified for area as surrogates for crypt and villus growth triggered by our treatments (Fig 1A and B). Freshly seeded mSIO crypts were grown for 96 h in control NR medium (Noggin, R-spondin1(R-SPO1)) or NR medium supplemented with either EGF (50 ng/ml) or EREG (50 ng/ml). EGF and EREG, as high- and low-affinity ligands of EGFR, respectively, are known to induce receptor phosphorylation and endocytosis (Freed et al, 2017).

**Figure S1.**
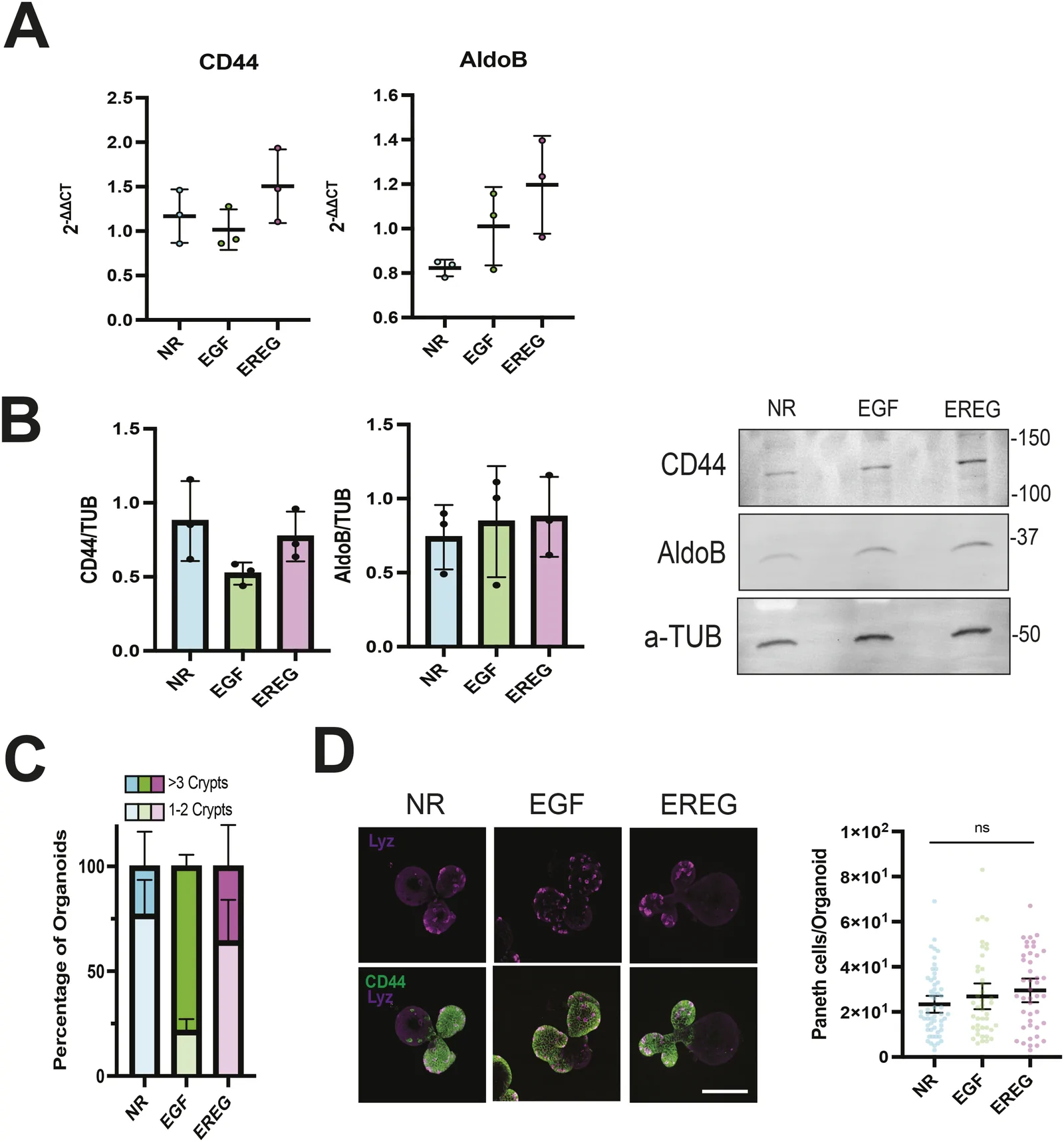
EGF and EREG do not alter Paneth cell differentiation or marker expression compared with NR media. **(A)** Organoids were grown with NR medium or supplemented with either EGF (50 ng/ml) or EREG (50 ng/ml) for 96 h with a complete medium change at 48 h. qRT-PCR of CD44 and AldoB, Results obtained from N = 3. Mean ± SD are shown. **(B)** **(**Left) Bar Graphs of mean **± **SD of CD44 and AldoB normalized by a-TUB. (Right) Immunoblot of CD44, AldoB and a-TUB with approximate MW in kD. **(C)** Percentage of organoids displaying either 1–2 or 3 or more crypts N = 3. **(D)** (Left) Organoids were immuno-stained with CD44 (Green) and lysozyme (Lyz, Magenta). Scatter plots showing mean ± 95% CI of Paneth cell number per organoid were quantified in each condition. Quantification of n = 43–56 organoids/condition, N = 3; significance was calculated by Kruskal-Wallis with Dunn’s multiple comparisons test. *P*-values <0.05 are shown.

EGF and EREG addition resulted in significantly enhanced organoid growth compared with NR medium, however, Crypt Area (CD44) was differentially enhanced by EGF, while EREG enhanced Crypt and Villus area (ALDO) (Fig 1C). The relative ratio of Crypt-to-Villus area (CD44/ALDO) showed that EGF strongly favored the Crypt proliferation over Villus differentiation, while NR medium and EREG maintained balanced development of Crypt and Villus, despite the overall larger size of EREG treated organoids relative to NR treatment (Fig 1C and D). Protein and mRNA expression levels of CD44 and AldoB did not show any significant changes upon treatments (Figs S1A and B and S8A), as these markers have fundamentally a very heterogeneous expression that varies from cell to cell depending on cell age or cell cycle status (Gracz et al, 2013; Zheng et al, 2023).

Crypt number was also increased by EGF, while EREG only had a small effect on the fraction of organoids with elevated number of crypts relative to NR organoids (Fig S1C). Crypt establishment is a direct result of apical constriction by Paneth cells (Langlands et al, 2016). Paneth cells are found in the crypt base, interspaced between ISCs and their abundance is often correlated to ISCs numbers (Tallapragada et al, 2021). Immunofluorescence staining showed that neither EGF nor EREG increased the number of Paneth cells, despite of the effect observed in the crypt area (Fig S1D).

These results suggest that both EGF and EREG can induce organoid development by differentially regulating the proliferation/differentiation balance, with EGF promoting CD44+ crypt enlargement rather than differentiation into the AldoB+ villus region. On the other hand, EREG promotes growth of both crypt and villus regions, thus suggesting that EGF-family ligands promote mSIOs development through distinctive downstream mechanisms.

### EGF- and EREG-mediated endocytosis reduces EGFR PM localization and expression

**Figure 2.**
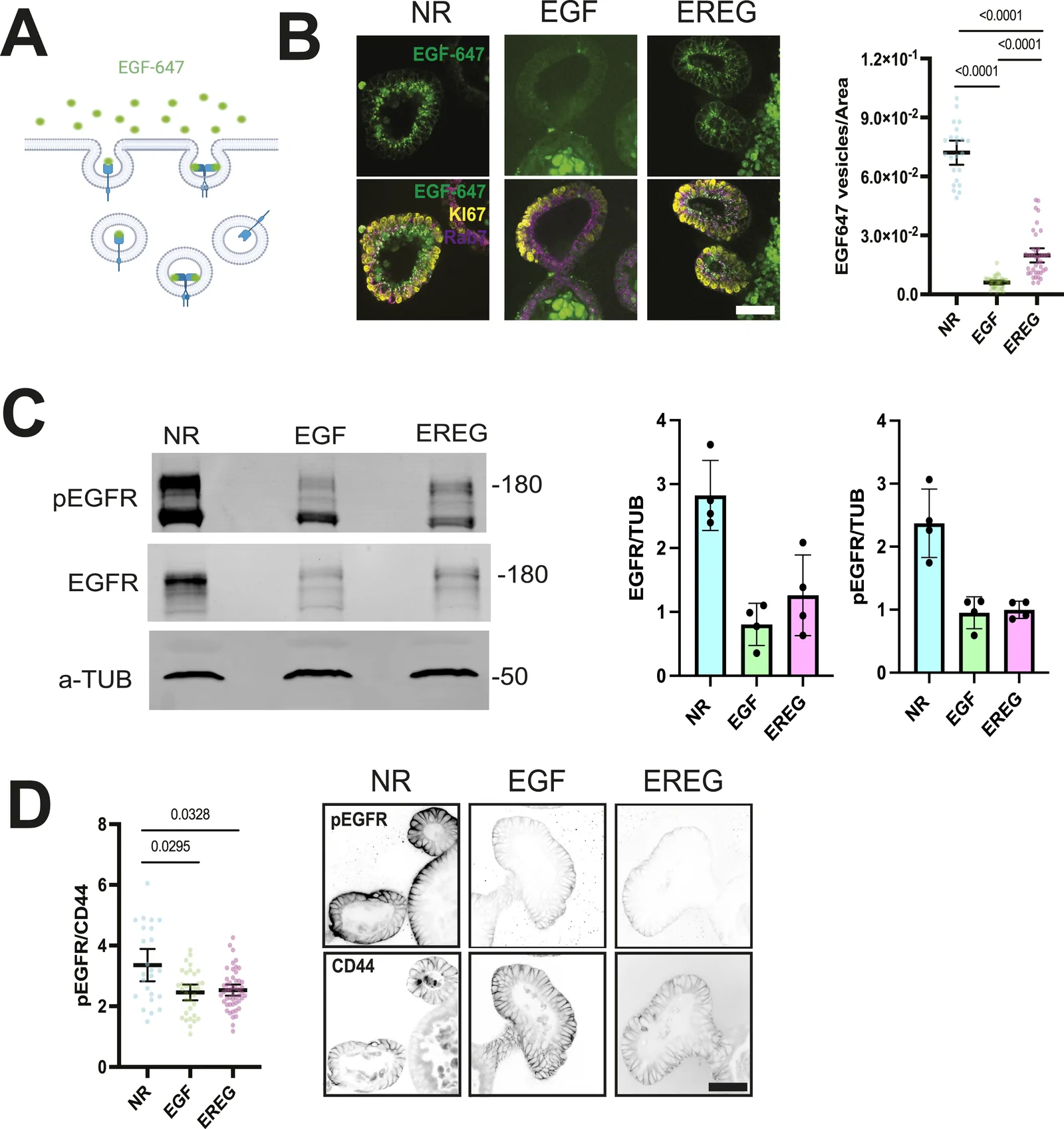
Plasma membrane localization of EGFR is reduced in organoids grown in the presence of EGF and EREG. **(A)** Alexa Fluor-647-labeled EGF (50 ng/ml) is added to organoids after 96 h of growth; after 30 min of incubation, organoids were fixed and stained. Alexa Fluor 647-positive endosomes indicate the level of surface EGFR and subsequent endocytosis upon ligand binding. **(B)** (Left) Representative micrographs of organoids after immunofluorescence staining against Ki67 (Yellow) and Rab7 (Magenta). EGF-647 internalized through EGFR endocytosis is labeled in (Green). Scale bar 20 μm. (Right) EGF-647 vesicles per area as scatter plots, mean ± 95% CI; N = 3, n = 28–37 crypts/condition; significance was calculated using the Kruskal-Wallis test with Dunn’s multiple comparisons test. *P*-values <0.05 are shown. **(C)** (Left) Immunoblot of pEGFR, EGFR and a-TUB with approximate MW in kDa. (Right) Bar Graphs of mean ± SD of pEGFR or EGFR relative to a-TUB. N = 4. **(D)** (Left) Ratio of pEGFR to CD44. Scatter plots of mean ± 95% CI. N = 3, n = 32–60 crypts/condition; significance was calculated by Kruskal-Wallis with Dunn’s multiple comparisons test. *P*-values <0.05 are shown. (Right) Representative micrographs of organoids after immunofluorescence staining against pEGFR (Top) and CD44 (Bottom). Scale bar 20 μm.

**Figure S2.**
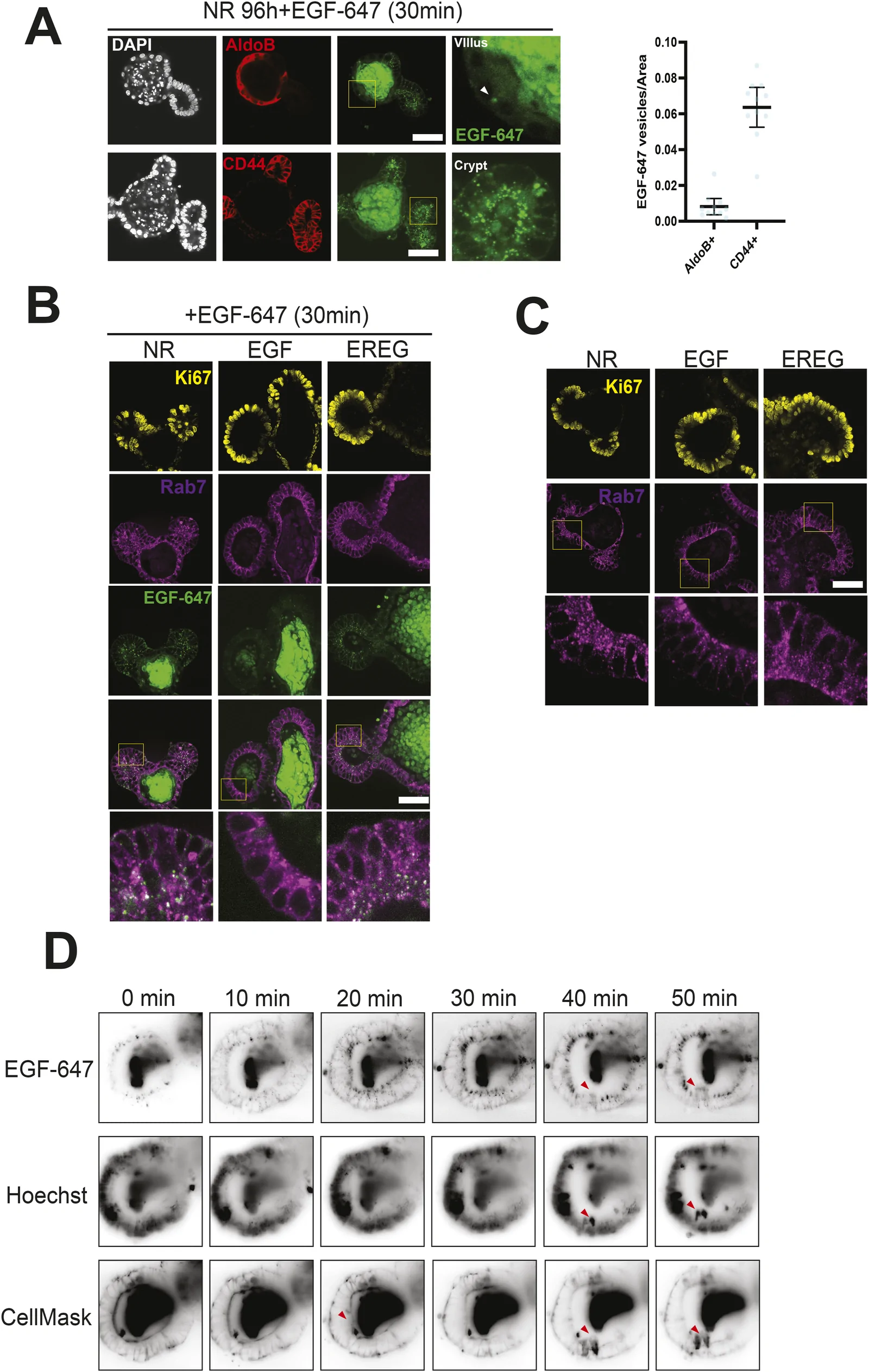
EGF-647 endocytosis in organoid crypts co-localizes with late endosomal marker Rab7. **(A)** Organoids were grown with NR medium and immuno-stained with DAPI (white) CD44 (Bottom panel, Red) or AldoB (Top panel, Red). 50 ng/ml of Alexa Fluor 647-labeled EGF (green) was added to organoids after 96 h of growth; after 30 min of incubation, organoids were fixed and stained. Yellow boxes denote zoom-in areas shown in the fourth column. Single endosomal vesicle in AldoB+ area (White triangle). **(B, C)** Organoids were grown with NR medium or supplemented with either EGF (50 ng/ml) or EREG (50 ng/ml) for 96 h with a complete medium change at 48 h. Organoids were immuno-stained for Ki67 (Yellow) and Rab7 (Magenta); yellow boxes denote zoom-in areas shown in the row below. **(B)** 50 ng/ml of Alexa Fluor 647-labeled EGF (Green) was added to organoids after 96 h of growth; after 30 min of incubation, organoids were fixed and stained. Yellow boxes denote zoom-in areas shown in the row below with EGF-647 (Green) and Rab7 (Magenta). **(D)** Digital light-sheet microscopy was used to see EGF-647 endocytosis in an organoid crypt grown for 96 h in NR medium. Images showing Cell Mask, Hoechst, and EGF-647 every 10 min. Red triangles show cells protruding into the lumen. For more details, see *Materials and Methods* and (Video 1).

EGF and EREG are known to bind EGFR and subsequently induce phosphorylation and endocytosis of EGFR. EREG is also a ligand of ERBB4; however, expression of this receptor is not detected in small intestinal epithelia or stroma (Lemmetyinen et al, 2023). In organoid culture, saturating concentrations of EGF are commonly used (Shankaran et al, 2021), reflecting conditions at which most of the PM-EGFR is internalized and undergoes lysosomal degradation, as demonstrated in cultured immortalized cells (Brüggemann et al, 2021). In contrast, EREG binding to EGFR promotes recycling back to the PM (Roepstorff et al, 2009). To quantify end-point surface levels of EGFR, organoids were incubated for 30 min with 50 ng/ml of EGF-647 before fixation and staining (Fig 2A), enabling quantification of the EGF-647 uptake and subsequent conclusion over PM EGFR abundance. We observed that most of the internalized EGF-647 was located in the crypt regions with few vesicles being observable in the villus regions (Fig S2A), this is consistent with the literature stating that EGFR is highly expressed in cycling cells within the intestinal crypt (Yang et al, 2017). EGF-647 endocytosis was observed in organoids grown in NR or EREG medium while organoids grown in presence of unlabeled EGF for 96 h showed very low endocytosis, suggesting reduced PM EGFR levels (Fig 2B). Absence of exogenous ligands likely allowed for accumulation of PM EGFR throughout the 96 h developmental window, while EREG medium promoted recycling to the PM, thereby maintaining high surface levels. As for organoids grown in EGF medium, it is likely that highly saturating EGF concentrations during 96 h were sufficient to completely deplete the cell surface of receptors, thus greatly reducing availability of PM EGFR for EGF-647 endocytosis.

Endocytosed EGF-647 co-localized with late endosome marker Ras-related protein Rab7 (Rab7), (Figs 2B and S2B and C). DLS (Digital Lightsheet) imaging of EGF-647 endocytosis shows an initial association with the crypt basal membrane, followed by the entry of vesicles that travel along the inner membranes towards the apical side (Fig S2D and Video 1). While basal to apical trafficking is clearly observed we cannot discard the possibility of apical endocytosis of EGF-647 from the lumen, as cell extrusions within the crypts are observable in the time frame of our experiments which could allow EGF-647 to seep into the luminal compartment (Fig S2D and Video 1). Transcytosis of EGF from basal to apical has been proposed in MDCK epithelial layers (Brändli et al, 1991), however little is known of the mechanisms involved.

Video 1 EGF-647 endocytosis in NR grown organoid crypt using Digital Light Sheet imaging. Panel showing time-lapse acquisition of EGF-647 endocytosis for 52 min after addition of ligand. Timestamp in minutes (min) in the upper-left corner. EGF-647 (Left panel), Hoechst (Middle panel) and CellMask (Right panel). Download video

As a PM receptor, EGFR phosphorylation is highly dependent on its interaction with ligands in the extracellular media; nevertheless, gene amplification enhancing expression levels or point mutations affecting inhibitory mechanisms can lead to EGFR phosphorylation in the absence of ligands (Arteaga & Engelman, 2014; Stanoev et al, 2018). Immunoblotting of both total and phosphorylated EGFR Y1068 showed that EGF- and EREG-grown organoids exhibited a significant reduction in EGFR protein expression compared with NR-grown organoids (Figs 2C and S8B). Quantification of Immunofluorescence staining for pEGFR Y1068, normalized to CD44 membrane staining, showed increased phosphorylation in crypts grown without EGF or EREG, whereas organoids grown with either ligand showed reduced reduced pEGFR Y1068 levels (Fig 2D).

EGFR endocytic cycle is fundamental to regulate receptor phosphorylation, allowing for degradation in late endosomes or interaction with protein tyrosine phosphatases (PTPs) within endomembrane compartments (Baumdick et al, 2015; Stanoev et al, 2018; Joshi et al, 2023). In turn, NR-grown organoids show an accumulation of pEGFR, likely due to endogenously expressed EGF or other ligands. Both EGF and EREG reduced total and surface expression levels of EGFR, and it is likely that the time frame between media changes every 48 h allows for a recovery of EGFR at the PM, thus rendering developing organoids competent to sense ligands in the environment.

### Temporal modulation of EGF-family ligands during development alters villus differentiation

**Figure S3.**
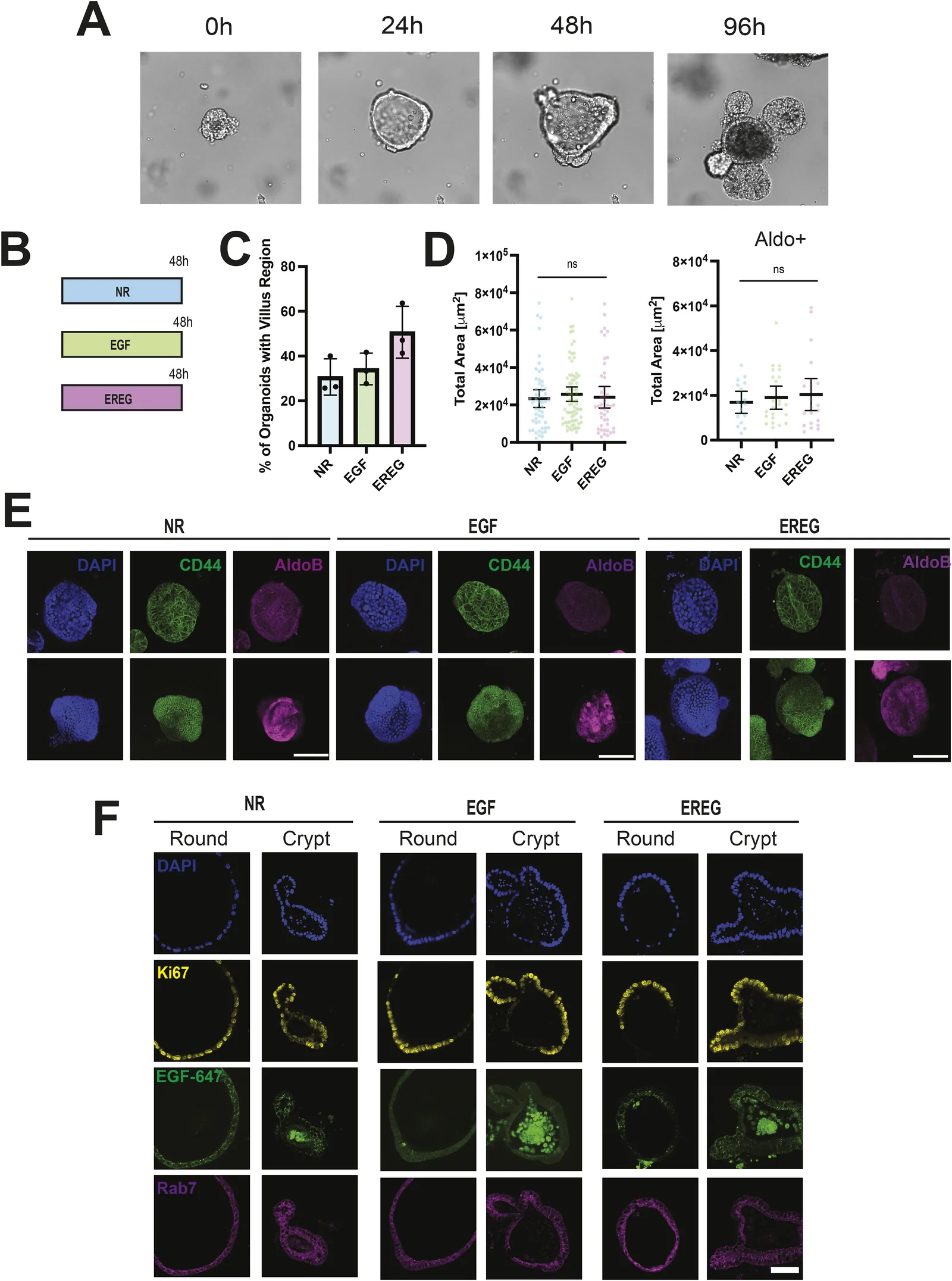
Organoids grown for 48 h often display no villus region and retain EGF-647 endocytosis in proliferating cells. **(A)** Brightfield time-lapse imaging of a developing organoid from seeded single crypts in EGF medium showing standard developmental trajectories between seeding (0 h), 24, 48, and 96 h. **(B)** Organoids were grown with NR medium or supplemented with either EGF (50 ng/ml) or EREG (50 ng/ml) for 48 h before fixing and staining. **(C)** Percentage of Organoids showing ALDO+ villus region after 48 h of development, N = 3. **(D)** Scatter plots of masked areas corresponding to total area, for all organoids (Left) and organoids with ALDO+ region showing mean ± 95% CI, N = 3, n = 45–70 organoids/condition (Right). Quantification and significance were calculated by the Kruskal-Wallis with Dunn’s multiple comparisons test. *P*-values <0.05 are shown; ns, non-significant. **(E)** Organoids were immuno-stained with CD44 (green), AldoB (magenta), and DAPI (blue). Organoids ALDO- (Top) and ALDO+ (Bottom) are shown for each treatment. Scale Bar 100 μm. **(F)** Alexa Fluor 647-labeled EGF (50 ng/ml) is added to organoids after 48 h of growth; after 30 min of incubation, organoids were fixed and stained with Ki67 (yellow), Rab7 (magenta), and DAPI (blue). EGF-647 is shown in (green). Representative images of round or crypt morphologies are shown for each treatment.

When developing from single crypts, organoids activate a regeneration program that initially drives proliferation into a fetal-like state where differentiation and stem cells markers are mostly absent (Sprangers et al, 2021). Budding within the organoid usually appears around the 48 h mark, where mature crypts start to develop (Sato et al, 2009; Fig S3A). After 48 h in culture, most organoids feature a round morphology that is entirely CD44 positive (Fig S3E), consistent with an undifferentiated, proliferating organoid (Serra et al, 2019). About 30% of them showed regions positive for AldoB staining, suggesting initial establishment of a villus region (Fig S3B and C). The percentage of organoids showing AldoB staining increases with EREG (Fig S3C) compared with other treatments, as shown previously for 96 h organoids grown with EREG (Fig 1C). Overall, the total area of organoids at 48 h did not change in any treatment condition, regardless of the presence or absence of AldoB staining (Fig S3D and E). After 48 h in culture, EGF-647 endocytosis was also clearly observed in NR and EREG-grown organoids but not in EGF-grown organoids (Fig S3F). Endocytosis was observed in organoids displaying either crypt or round morphology, often confined to proliferating cells, as indicated by Ki67 staining (Fig S3F).

In vitro cell culture conditions often promote proliferation that ultimately allows biomass expansion required for biochemical assays, and organoid culture is no exception. In the conception of ENR medium, addition of EGF and R-SPO1 was sufficient to induce organoid growth from single crypts, and the addition of Noggin as Bone morphogenetic protein (BMP) inhibitor proved instrumental for stemness maintenance and organoid passaging over time (Sato et al, 2009). While lower concentrations of EGF (e.g., 10 ng/ml) were also tested in this seminal work with similar results (Sato et al, 2009), little consideration was given to organoid developmental pathways in response to the evolving availability of EGF with regard to ligand concentration or stimulus duration.

**Figure 3.**
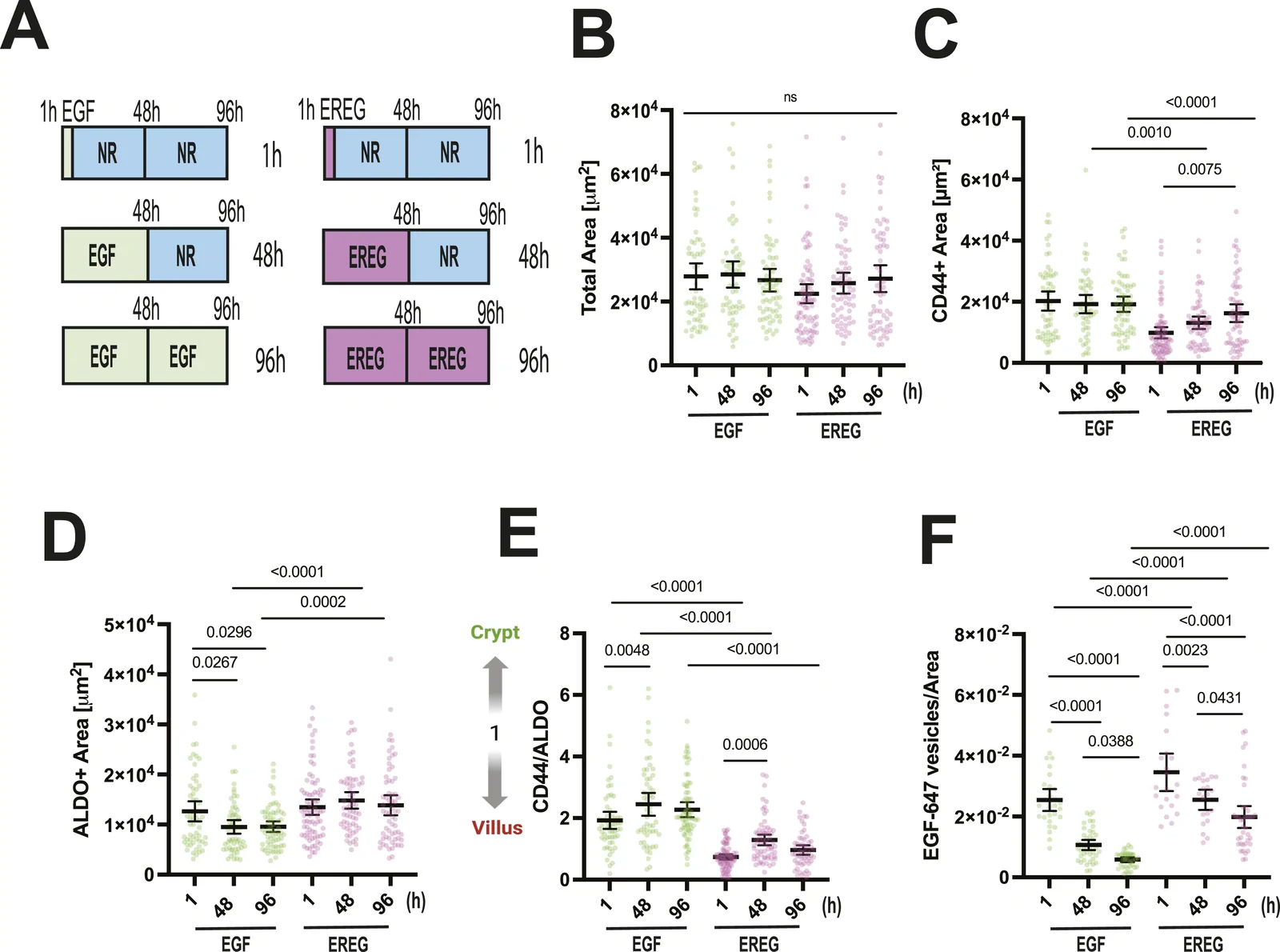
Removing EGF or EREG after crypt seeding does not modify crypt and villus development. **(A)** Organoids in (B, C, D, E, F) were grown with NR medium supplemented with either EGF (50 ng/ml) or EREG (50 ng/ml) for 48 h or 1 h, then complete medium was replaced with NR medium until fixation at 96 h. **(B, C, D)** Scatter plots showing mean ± 95% CI, N = 3, n = 57–79 organoids/condition of masked total, CD44+ and ALDO+ areas. Significance was calculated using two-way ANOVA and Sidak’s multiple comparisons test. *P*-values <0.05 are shown. **(E)** Relative ratio of CD44 to ALDO areas from (C) and (D). Scatter plot mean ± 95% CI of relative ratio within CD44/ALDO areas. Values above 1 suggest organoid development tends towards crypt proliferation, while values below 1 tend to villus differentiation. Significance was calculated by Two-way ANOVA and Sidak’s multiple comparisons test. *P*-values <0.05 are shown. **(F)** EGF-647 vesicles per area, shown as scatter plots with mean ± 95% CI; N = 3, n = 28–41 crypts/condition; significance was calculated by Two-way ANOVA and Sidak’s multiple comparisons test. *P*-values <0.05 are shown.

To test the temporal requirements of EGF/EGFR signaling in organoid development, freshly seeded single crypts were grown for 96 h with either EGF or EREG. However, a complete medium exchange with NR medium (No EGF or EREG) was done after 1 h and/or after 48 h of incubation (Fig 3A).

**Figure S4.**
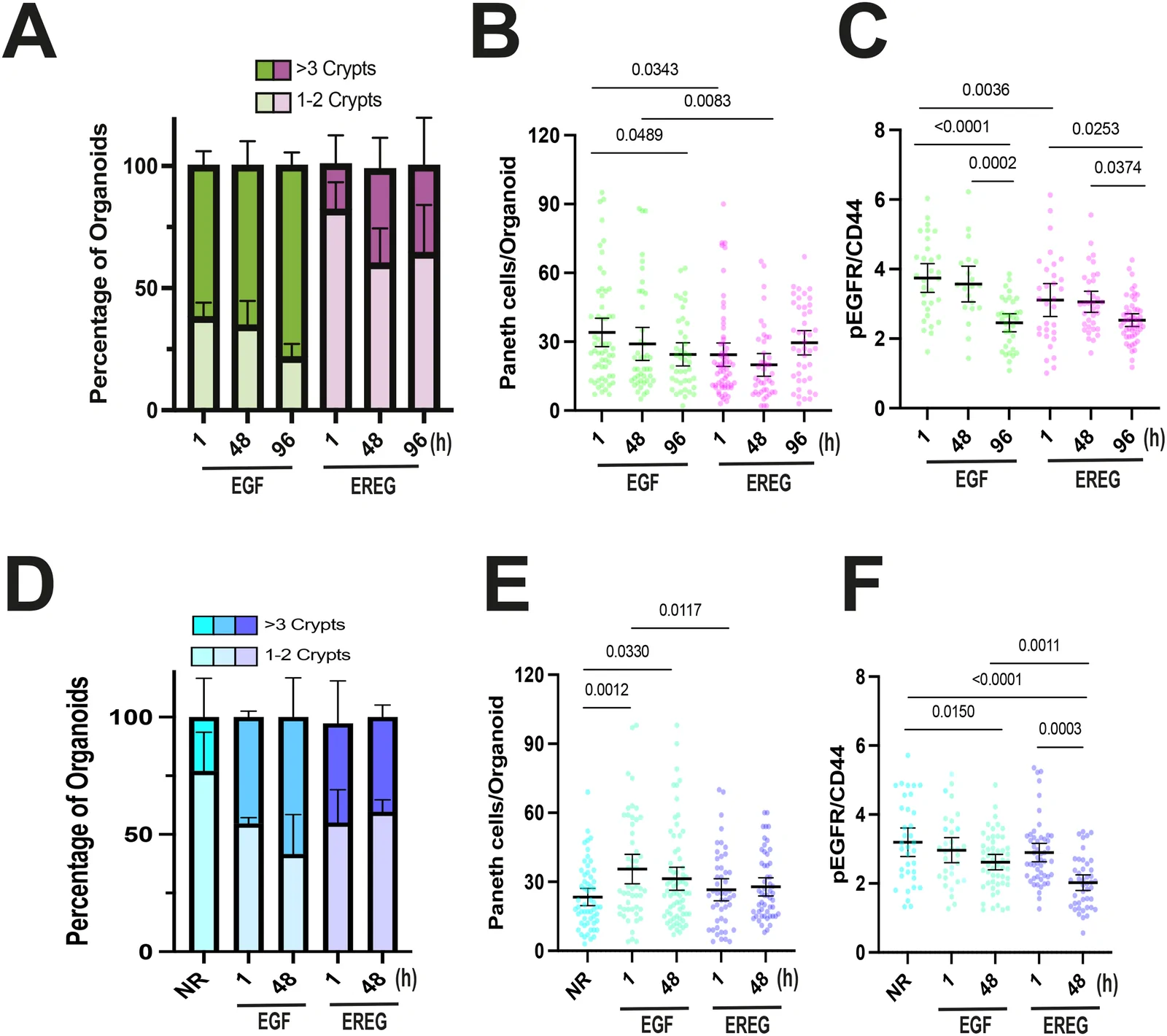
Effect of EGF/EREG temporal modulation in Crypt number, Paneth cell number, and pEGFR staining. **(A, B, C)** Organoids were grown with NR medium supplemented with either EGF (50 ng/ml) or EREG (50 ng/ml) for 48 h or 1 h, then complete medium was replaced with NR medium until fixation at 96 h. **(A)** Percentage of organoids displaying either 1–2 or 3 or more crypts N = 3. **(B)** Scatter plots showing mean ± 95% CI of Paneth cell number per organoid were quantified in each condition. Quantification of n = 42–61 organoids per condition, N = 3; significance was calculated by two-way ANOVA and Sidak’s multiple comparisons test. *P*-values <0.05 are shown in (C). Ratio of pEGFR to CD44. Scatter plots of mean ± 95% CI. N = 3, n = 21–49 crypts/condition; significance was calculated by two-way ANOVA and Sidak’s multiple comparisons test. *P*-values <0.05 are shown. **(D, E, F)** Organoids were grown for 1 and 48 h with NR, then the complete medium was replaced with NR medium supplemented with either EGF (50 ng/ml) or EREG (50 ng/ml). **(D)** Percentage of organoids displaying 1–2 or 3+ crypts N = 3. **(E)** Scatter plots showing mean ± 95% CI of Paneth cell number per organoid were quantified in each condition. Quantification of n = 50–69 organoids/condition, N = 3; significance was calculated by two-way ANOVA and Sidak’s multiple comparisons test. *P*-values <0.05 are shown. **(F) **Ratio of pEGFR to CD44. Scatter plots of mean ± 95% CI. N = 2–3, n = 39–54 crypts/condition; significance was calculated by Two-way ANOVA and Sidak’s multiple comparisons test. *P*-values <0.05 are shown.

**Figure S5.**
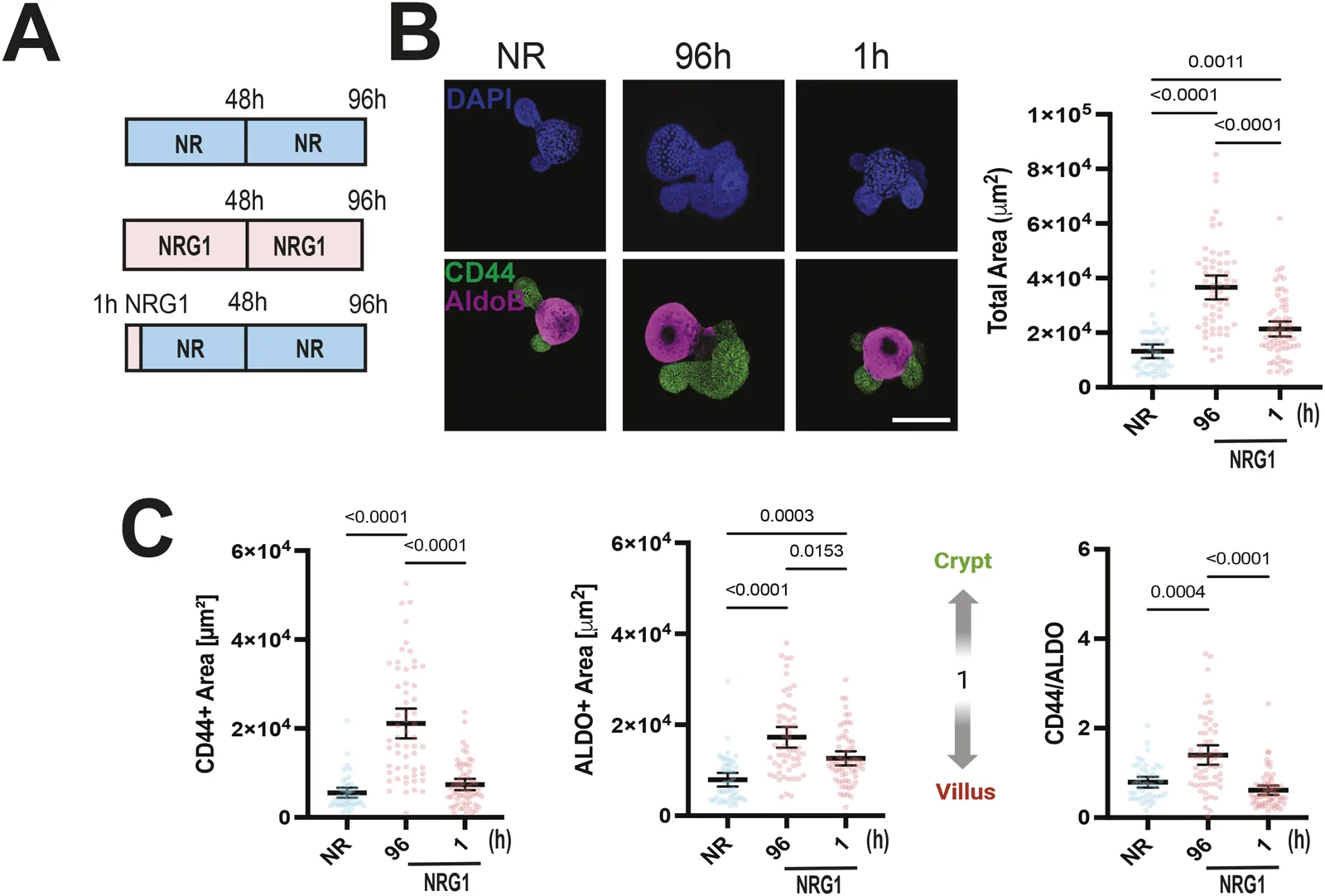
A brief 1 h NRG1 treatment is insufficient to induce long-term organoid development. **(A)** Organoids were grown in NR medium or NR medium supplemented with NRG1 (50 ng/ml) for 96 h or 1 h. After 1 h complete medium was replaced with NR medium until fixation at 96 h. **(B)** Organoids were immuno-stained with CD44 (Green), AldoB (Magenta), and DAPI (Blue). Scale Bar 100 μm. **(B, C)** Scatter plots showing mean ± 95% CI of masked areas corresponding to Total, CD44+, ALDO+, and ratio Quantification of n = 58–65 organoids/condition, N = 2–3; significance was calculated by the Kruskal-Wallis with Dunn’s multiple comparisons test. *P*-values <0.05 are shown.

Regardless of EGF or EREG removal time-frame, organoids showed comparable total area after 96 h in culture (Fig 3B). Moreover, 1 and 48 h of EGF-containing medium were sufficient to induce crypt growth comparable to 96 h with constant EGF (Fig 3C and E). While EGF withdrawal treatments were successful in maintaining Crypt development, EREG removal maintained villus differentiation size comparable to that of the 96 h treatment (Fig 3D and E). Surprisingly, 1 h EGF treatment not only induced crypt growth, but also enhanced villus differentiation to levels similar to those observed in EREG treatment (Fig 3D and E). Removal of EGF or EREG did not affect crypt number; however, removal of EGF increased Paneth cell number compared with the 96 h control (Fig S4A and B). Alternatively, seeded single crypts were treated for 96 h or 1 h with 50 ng/ml of NRG1 (Fig S5A), an EGF-family ligand known to bind ERBB3 and ERBB4 but not EGFR (Fock et al, 2015). NRG1 was able to induce organoid growth as previously shown (Jardé et al, 2020; Lemmetyinen et al, 2023). Notably, 1 h treatment with NRG1 and subsequent withdrawal failed to induce organoid growth to 96 h levels, but still showed a significant growth increase relative to NR-grown organoids (Fig S5B and C). Therefore, these robust signaling responses under EGF and EREG treatments are likely mediated by EGFR.

96 and 48 h incubation with EGF led to a near-complete depletion of surface EGFR, as very low EGF-647 endocytosis was detected (Fig 3F). In contrast, 1 h incubation with EGF exhibited high endocytic activity, suggesting high surface EGFR levels, similar to EREG treatments, which increased endocytic activity over increasing EREG-starvation times (Fig 3F). EGFR phosphorylation was enhanced by EGF or EREG removal compared with the 96-h treatments, further highlighting that phosphorylation of EGFR requires PM localization (Fig S4C).

**Figure 4.**
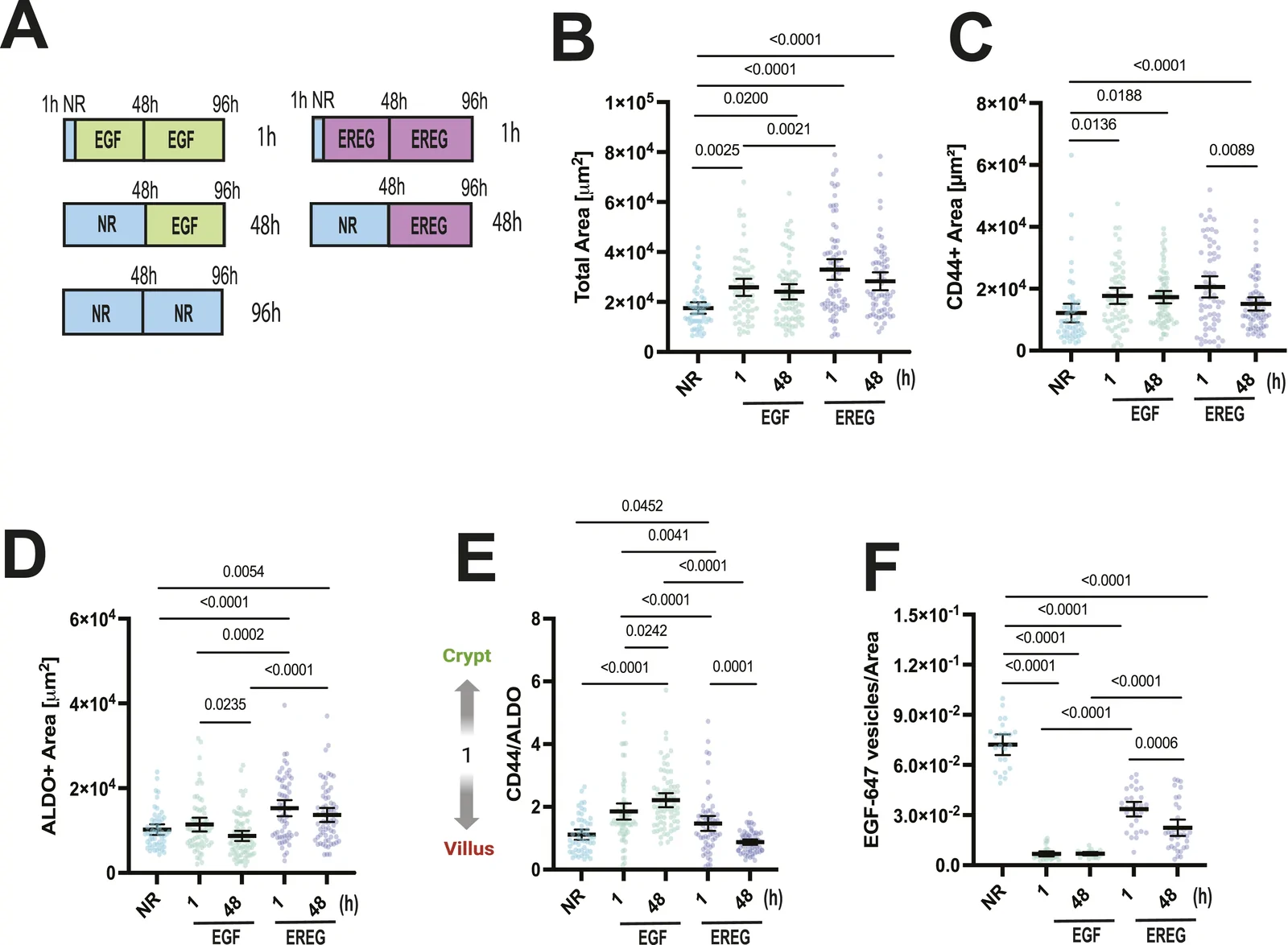
Introducing EGF and EREG later in mSIO development triggers crypt and villus development, similar to early treatment. **(A)** Organoids in (B, C, D, E, F) were grown for 1 and 48 h with NR, then complete medium was replaced with NR medium supplemented with either EGF (50 ng/ml) or EREG (50 ng/ml). **(B, C, D)** Scatter plots showing mean ± 95% CI, N = 3, n = 57–73 organoids/condition of masked total, CD44+ and ALDO+ areas. Significance was calculated using two-way ANOVA and Sidak’s multiple comparisons test. *P*-values <0.05 are shown; ns, non-significant. **(E)** Relative ratio of CD44 to ALDO areas from (C) and (D). Scatter plot mean ± 95% CI of relative ratio within CD44/ALDO areas. Values above 1 suggest organoid development tends towards crypt proliferation, while values below 1 tend to villus differentiation. Significance was calculated using two-way ANOVA and Sidak’s multiple comparisons test. *P*-values <0.05 are shown. **(F)** EGF-647 vesicles per area, shown as scatter plots with mean ± 95% CI; N = 3, n = 20–38 crypts/condition; significance was calculated by two-way ANOVA and Sidak’s multiple comparisons test. *P*-values <0.05 are shown.

**Figure S6.**
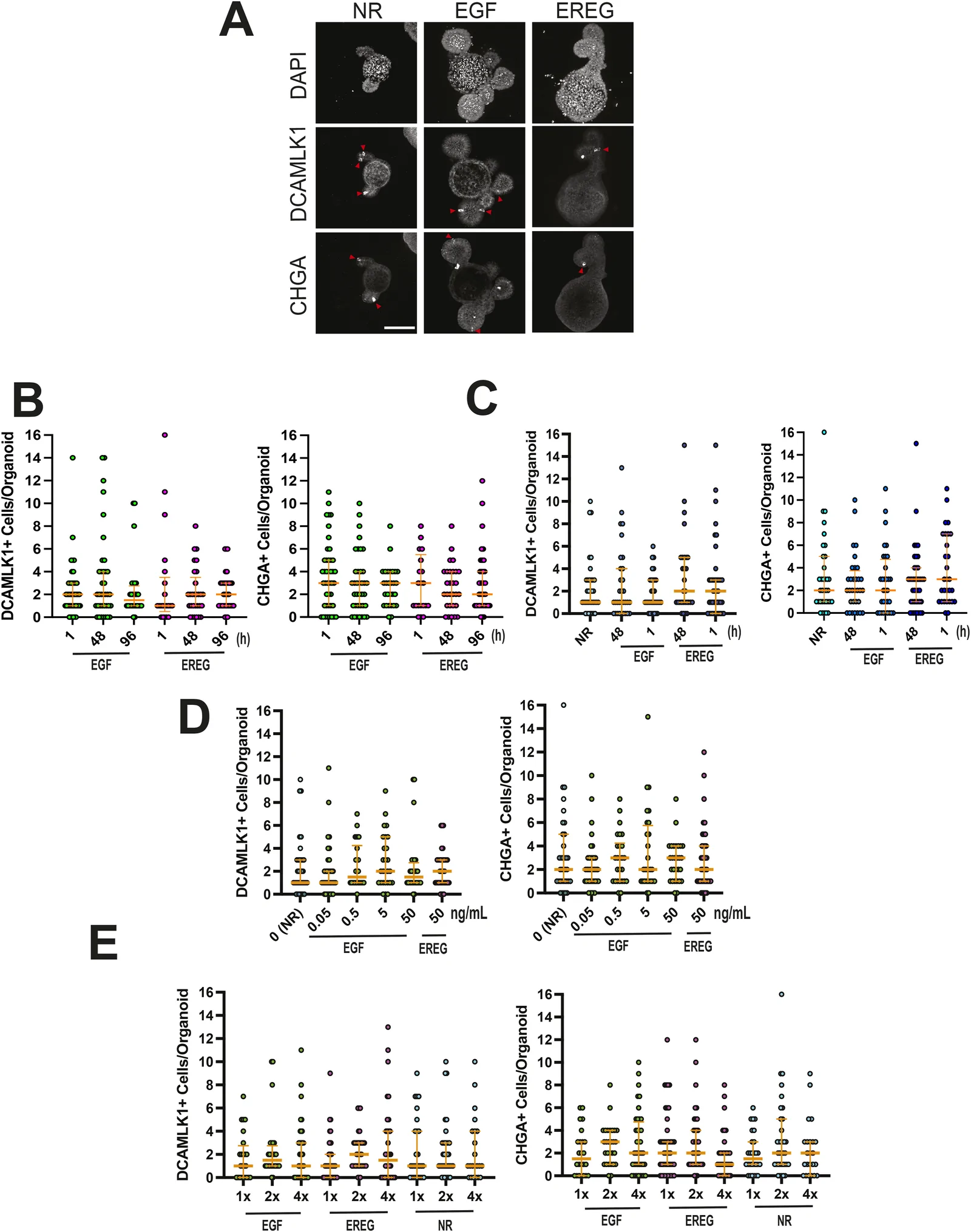
CHGA+ and DCAMKL1+ entero-endocrine cells are not affected by ligand regulated EGFR plasma membrane localization. **(A)** Organoids were fixed 96 h after seeding and immuno-stained with CHGA, DCAMKL1, and DAPI. Organoids were grown as described in Fig 1; representative organoids for NR, EGF, and EREG are shown. Grayscale Maximum intensity projections are shown. Scale Bar 100 μm. **(B, C, D, E)** Quantification of CHGA+ and DCAMKL1+ cells per organoid across different media treatment conditions. Orange bars show the median with interquartile range. **(B)** Organoids were grown with NR medium supplemented with either EGF (50 ng/ml) or EREG (50 ng/ml) for 48 h or 1 h, then complete medium was replaced with NR medium until fixation at 96 h as described in Fig 3. Quantification obtained from n = 21–40 organoids collected in N = 2–3 experiments. Results were nonsignificant by two-way ANOVA and Sidak’s multiple comparisons test. **(C)** Organoids were grown for 1 and 48 h with NR, then the complete medium was replaced with NR medium supplemented with either EGF (50 ng/ml) or EREG (50 ng/ml) as described in Fig 4. Quantification obtained from n = 29–41 organoids collected in N = 3 experiments. Results were nonsignificant by two-way ANOVA and Sidak’s multiple comparisons test. **(D)** Organoids were grown with NR medium, supplemented with EGF at different concentrations: 50, 5, 0.5, and 0.05 ng/ml or EREG 50 ng/ml for 96 h before fixation and immunostaining as described in Fig 5. Quantification obtained from n = 24–36 organoids collected in N = 3 experiments. Results were nonsignificant by two-way ANOVA with Sidak’s multiple comparisons test. **(E)** Organoids were grown with NR medium or supplemented with either EGF (50 ng/ml) or EREG (50 ng/ml) for 96 h, with a complete medium change every 24 h (4×), 48 h (2×), or 96 h (1×), as shown in Fig 6. Quantification obtained from n = 24–36 organoids collected in N = 3 experiments. Results were nonsignificant by two-way ANOVA with Sidak’s multiple comparisons test.

In a complementary experiment, single crypts were seeded in NR medium, and ligands were reintroduced after 48 or 1 h of growth in NR medium (Fig 4A). Reintroducing EGF- and EREG-containing medium increased total organoid area and crypt area compared with continuous growth in NR medium (Fig 4B and C). In contrast to crypts treated with EREG from seeding (Fig 3C), adding EREG after growth factor starvation enhanced crypt growth, similar to EGF, and simultaneously increased villus growth (Fig 4C–E). Likewise, there was a marked increase in the number of organoids featuring multiple crypts, although only EGF treatment enhanced the number of Paneth cells (Fig S4D and E). Since EGFR has been shown to regulate the differentiation of DCAMKL1+ entero-endocrine cells (Basak et al, 2017), we stained organoids for the entero-endocrine markers chromogranin A (CHGA) and DCAMKL1. Discrete single cells could be easily identified after staining either in the villus or crypt regions; however, the number of cells did not change upon addition or withdrawal of EGF or EREG (Fig S6A–C).

Reintroduction of EGF and EREG into the culture medium greatly reduced endocytosis of labeled EGF-647, with EREG-treated organoids retaining some endocytic capacity consistent with EGFR recycling towards the PM (Fig 4F). EGF or EREG removal from the culture medium increased EGFR phosphorylation; likewise, reintroducing EGF or EREG at 48 h after seeding significantly reduced phosphorylation, whereas brief 1 h starvation did not change phosphorylation levels compared with the NR condition (Fig S4F).

Altogether, these results highlight the remarkable robustness of EGFR signaling in response to high-affinity and low-affinity ligands, an effect that could not be replicated by NRG1 treatment, which does not bind EGFR. EGF or EREG treatment at any tested time point reduced EGFR surface levels compared with NR and led to similar total area growth, with 1 h treatments upon seeding being sufficient to induce crypt and villus development for EGF and EREG treatments, respectively.

### Sub-saturating concentrations of EGF enhance both crypt proliferation and villus differentiation

**Figure 5.**
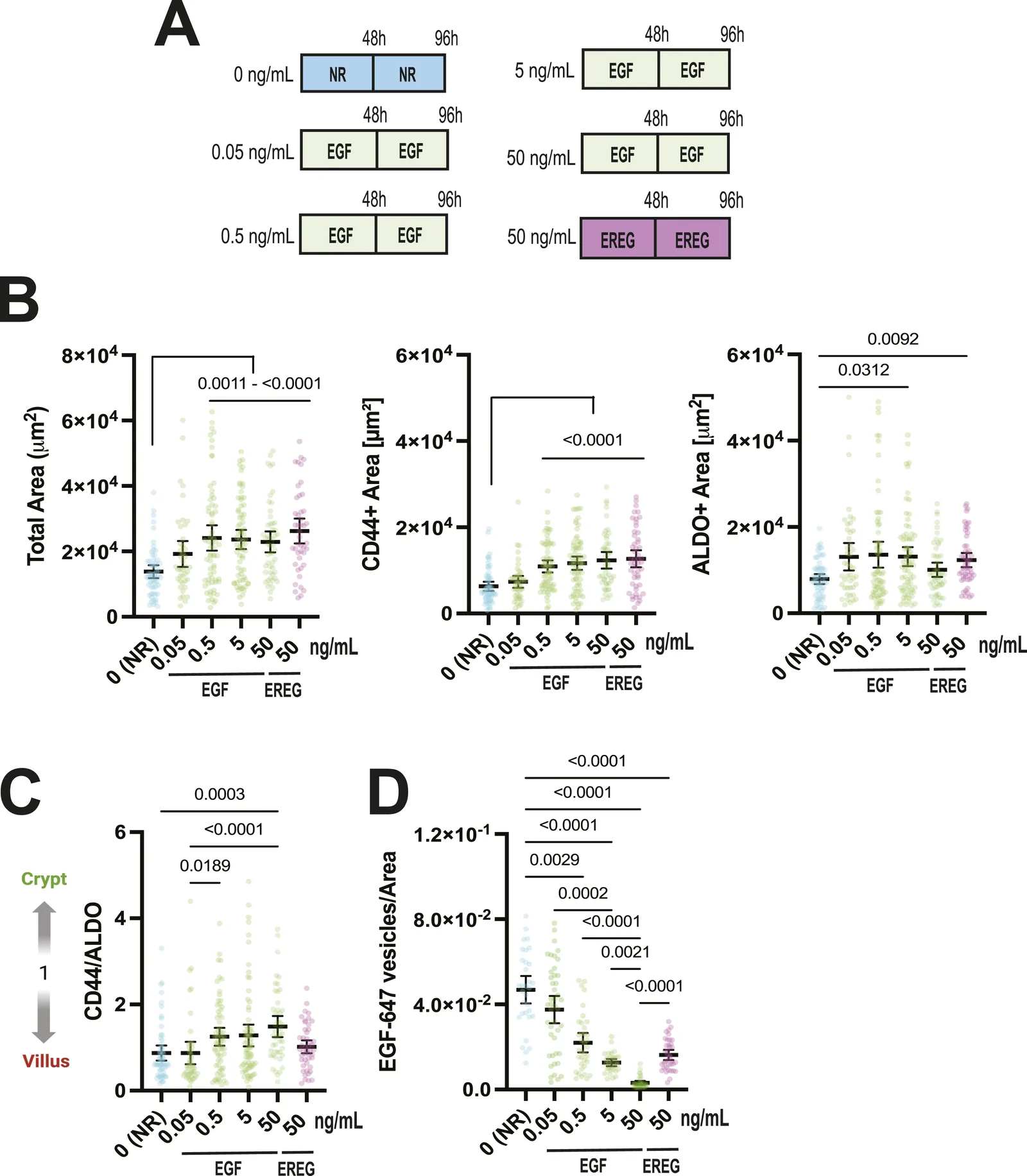
Sub-saturating concentrations of EGF trigger both crypt and villus growth by reducing EGFR expression at the PM. **(A)** Organoids in (B, C, D) were grown in NR medium supplemented with EGF at different concentrations (50, 5, 0.5, and 0.05 ng/ml) or with EREG at 50 ng/ml for 96 h before fixation and immunostaining. **(B)** Scatter plots showing mean ± 95% CI, N = 3, n = 44–71 organoids per condition of masked Total, CD44+ and ALDO+ areas; significance was calculated by Kruskal-Wallis with Dunn’s multiple comparisons test. *P*-values <0.05 are shown. **(C) **Relative ratio of CD44 to ALDO areas from (B). Scatter plot mean ± 95% CI of relative ratio within CD44/ALDO areas. Values above 1 suggest organoid development tends towards crypt proliferation, while values below 1 tend to villus differentiation. Significance was assessed using the Kruskal-Wallis test with Dunn’s multiple comparisons test. *P*-values <0.05 are shown. **(D)** EGF-647 vesicles per area, shown as scatter plots with mean ± 95% CI; N = 3, n = 39–48 crypts/condition; significance was assessed using the Kruskal-Wallis test with Dunn’s multiple comparisons test. *P*-values <0.05 are shown.

Given that organoids can develop in EGF-free medium, albeit with smaller crypt and villus sizes, and that EREG, a low-affinity ligand of EGFR, induces organoid development while maintaining high levels of surface EGFR, we tested whether lower, sub-saturating concentrations of EGF could sustain organoid growth. Freshly seeded single crypts were cultured for 96 h with EGF concentrations decreasing by one order of magnitude, starting at 50–0.05 ng/ml (Fig 5A). NR treatment (0 ng/ml EGF) showed consistent decreased growth compared with 50 ng/ml, however, concentrations in between showed marked differences in crypt and villus development.

Compared with NR, total and crypt growth was significantly enhanced starting at 0.5 ng/ml of EGF (Fig 5B). On the other hand, villus differentiation increased significantly from 5 ng/ml and then decreased at 50 ng/ml (Fig 5B). Likewise, the CD44/ALDO ratio favored crypt development starting at 0.5 ng/ml and maintained a similar ratio at higher EGF concentrations (Fig 5C). The number of CHGA+ or DCAMKL1+ entero-endocrine cells was unchanged by sub-saturating concentrations of EGF (Fig S6D).

EGF-647 endocytosis also showed a negative correlation between surface EGFR and medium EGF concentrations (Fig 5D). Notably, concentrations that significantly enhanced villus development at 5 ng/ml EGF showed surface EGFR levels similar to those of EREG treatment (Fig 5D).

**Figure S7.**
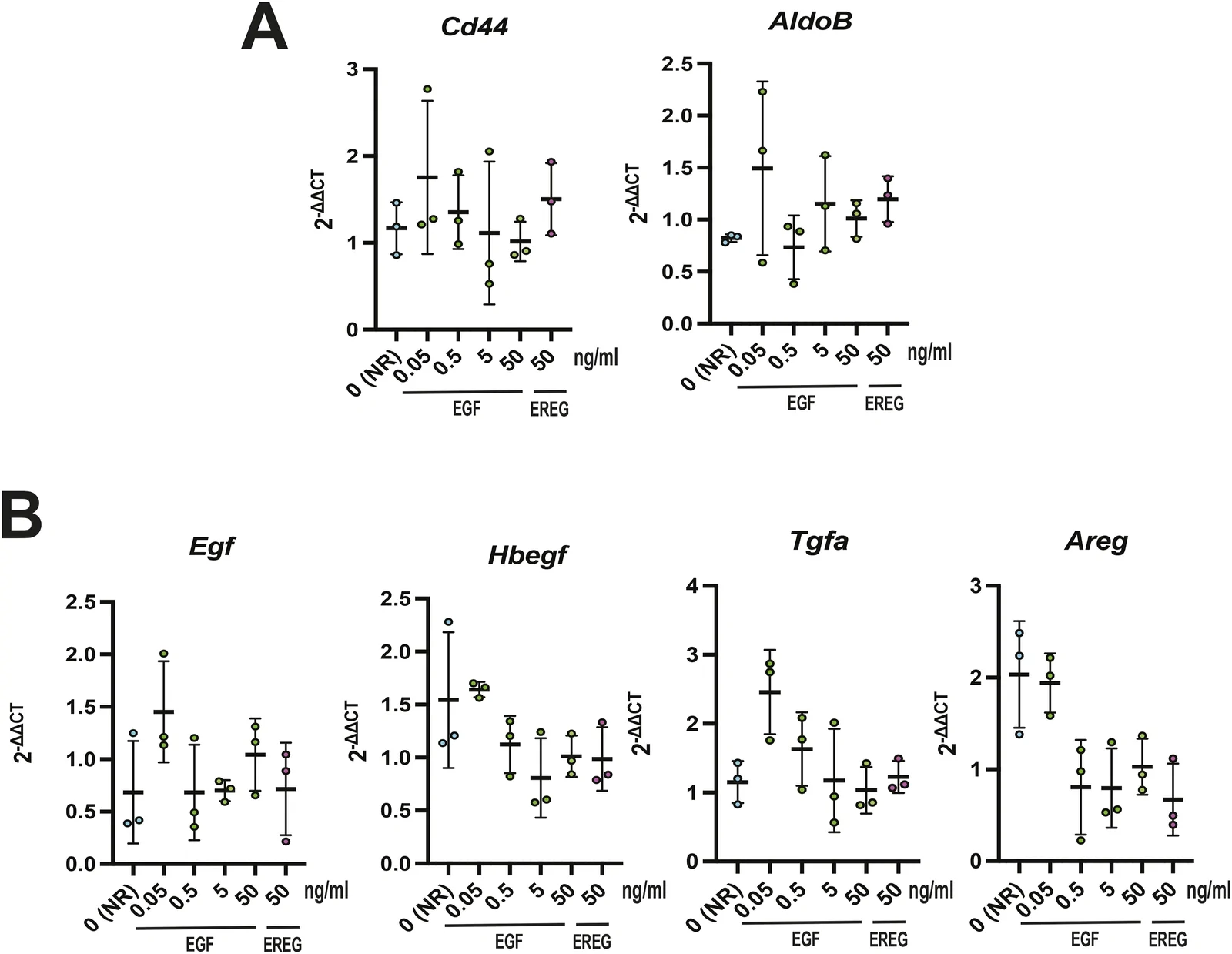
EGFR ligands are upregulated in organoids grown with sub-saturating amounts of EGF. **(A, B)** Organoids were grown in NR medium supplemented with EGF at different concentrations (50, 5, 0.5, and 0.05 ng/ml) or EREG at 50 ng/ml for 96 h before RNA extraction. **(A)** qRT-PCR quantification of *Cd44* and *AldoB* transcripts. Scatter plots showing Mean ± SD from N = 3 independent experiments. **(B)** qRT-PCR quantification of *Egf, Hbegf, Tgfa, Areg* transcripts. Scatter plots showing Mean ± SD from N = 3 independent experiments.

Limited availability of extracellular ligands may enhance the endogenous expression of other EGF-family ligands to maintain downstream signaling; furthermore, high and low doses of EGF have been shown to activate distinct transcriptional programs (Weisheng et al, 2025). qRT-PCR showed that while *Cd44* and *AldoB* expression is mostly unchanged (Fig S7A), EGFR ligands such as *Egf, Hbegf, Tgfa*, and Areg are differentially increased in NR media or in the presence of 0.05 ng/ml EGF (Fig S7B). Expression of these ligands may allow for basal EGFR activation to be maintained in the absence of media-supplemented EGF family ligands. Nevertheless, it does not seem that expression of these ligands ultimately modifies gross development of the crypt and villus regions and very likely is necessary to maintain the stem cell niche in absence of other ligands (Abud et al, 2021).

### Multiple pulses of EGF deplete EGFR surface expression reducing villus differentiation

Removing saturating amounts of EGF from the culture medium, EREG treatment, and sub-saturating EGF concentrations all maintain a high levels of pEGFR expression compared with long-term EGF treatment in saturating concentrations. A combination of biosynthesis and endocytic cycling is continually supplying EGFR to the cell surface; thus, this constant turnover is likely critical for the phenotypic outcomes we observe.

**Figure 6.**
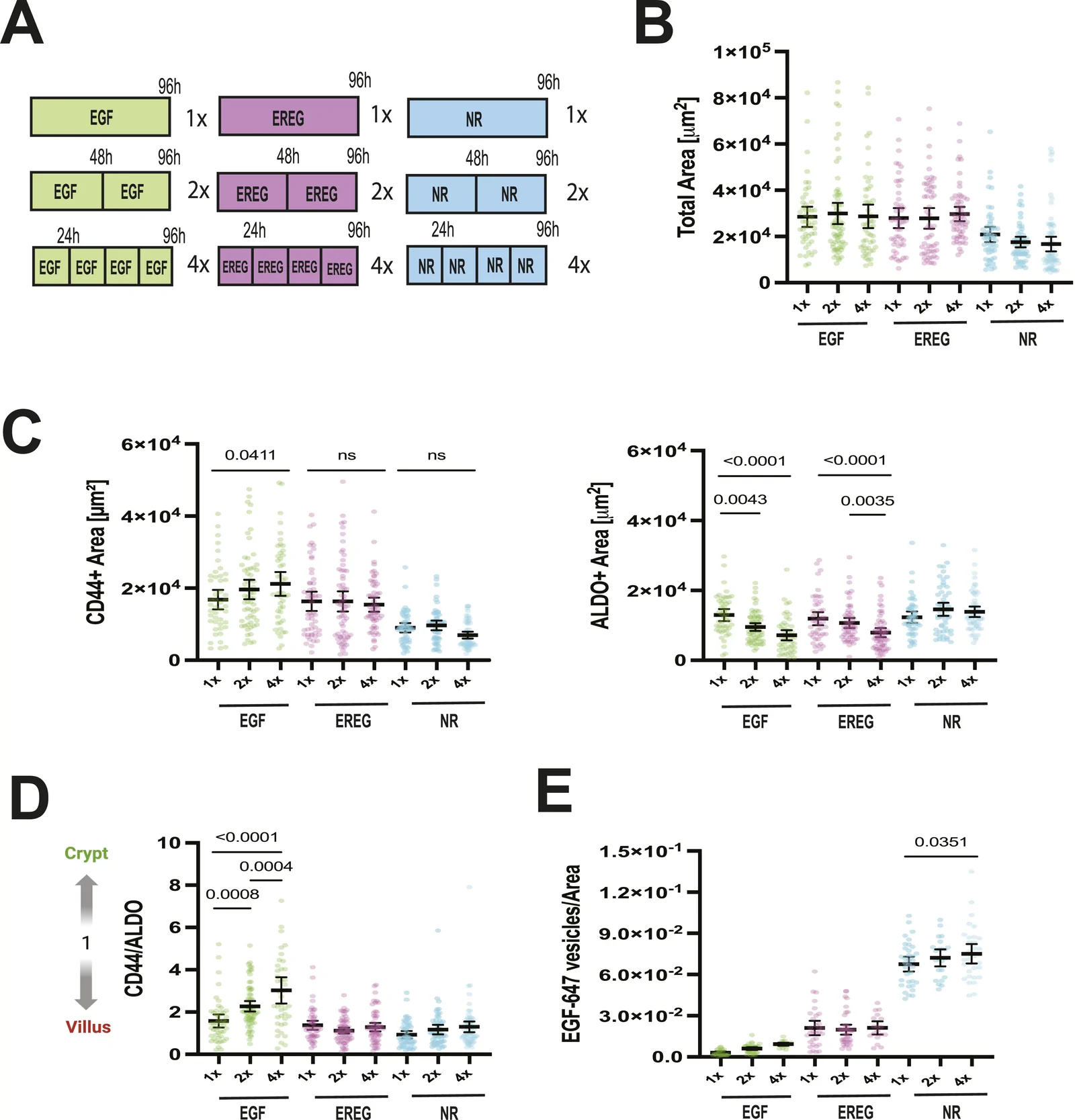
Multiple EGF or EREG medium changes reduce villus differentiation. **(A)** Organoids in (B, C, D, E) were grown in NR medium or supplemented with either EGF (50 ng/ml) or EREG (50 ng/ml) for 96 h with a complete medium change every 24 h (4×), 48 h (2×), or 96 h (1×). **(B, C)** Scatter plots showing mean ± 95% CI, N = 3, n = 47–67 organoids/condition of masked areas corresponding to (B) Total Area and (C) CD44+ and ALDO+ areas; significance was calculated by two-way ANOVA and Sidak’s multiple comparisons test. *P*-values <0.05 are shown. **(D)** Relative ratio of CD44 to ALDO areas from (C). Scatter plot mean ± 95% CI of relative ratio within CD44/ALDO areas. Values above 1 suggest organoid development tends towards crypt proliferation, while values below 1 tend to villus differentiation. Significance was calculated by two-way ANOVA and Sidak’s multiple comparisons test. *P*-values <0.05 are shown. **(E)** EGF-647 vesicles per area as scatter plots showing mean ± 95% CI; N = 3, n = 21–40 crypts/condition; significance was calculated by Two-way ANOVA and Sidak’s multiple comparisons test. *P*-values <0.05 are shown.

To further test this idea, freshly seeded single crypts were grown for 96 h; however, the fresh medium was provided only once, twice, or four times during this period to continuously deplete PM EGFR in developing organoids (Fig 6A). Total Area of the organoids remained unchanged in EGF and EREG, regardless of the number of medium changes, indicating that fresh medium addition does not alter overall organoid growth (Fig 6B). In contrast, crypt area increased while villus area decreased with multiple EGF medium changes, an effect only partially observed with EREG treatment, which maintained Crypt Area across all treatments while exhibiting decreasing Villus areas (Fig 6C). This change did not significantly affect Crypt/Villus balance (Fig 6D) or enteroendocrine cells development (Fig S6E). As expected, multiple EGF treatments greatly reduced EGF-647 endocytosis while EREG and NR treatments maintained similar levels of endocytosis in all treatments (Fig 6E), highlighting that regardless of the number of media changes, these treatments promote EGFR PM localization likely through recycling.

## Discussion

Human plasma levels of EGF range between 0.04 and 0.1 ng/ml (Alshammari et al, 2026), whereas concentrations of up to 300 ng/ml have been identified in rodent milk and are essential for early gastrointestinal development in newborns. After weaning and with age, EGF levels in the intestine are greatly reduced (Schaudies et al, 1989; Fisher & Lakshmanan, 1990). EGF expression is also up-regulated in Paneth cells upon injury in the intestinal epithelium (Calafiore et al, 2023). Also, other EGF-family ligands play a substantial role during damage response and regeneration processes in the intestine, including HB-EGF and AREG, highlighting the potential role of EGFR-binding ligands during organoid development (Shao & Sheng, 2010; Su et al, 2013). Absolute concentrations of EGF-family ligands in tissues could also be obscured by tissue geometry, such as the confined space within an intestinal crypt, allowing for a higher concentration of ligand within a lower volume (Tschumperlin et al, 2004; Schmick & Bastiaens, 2014). In all, the intestinal tissue is exposed to changing ligand concentrations throughout development, with higher levels often associated with epithelial regeneration processes interspersed with periods of low ligand availability, during which proliferation and differentiation are balanced. In turn, periods of low ligand availability enhance the long-term stability of EGFR expression and its enrichment at the PM, making intestinal crypts susceptible to changes in ligand concentration in the environment.

In the present work, we attempt to bridge the vast knowledge currently available on the role of EGF-family ligands in intestinal development with the dynamics of EGFR signaling and the endocytic cycle (Koseska & Bastiaens, 2020; Jendrisek et al, 2026). To test this, we systematically added and removed EGF and EREG, quantified the amount of EGFR at the PM after treatments, and ultimately assessed the phenotypic effects on organoid development. CD44 and AldoB were used as markers for the proliferative crypt region and the differentiated villus region in mSIOs. We chose these markers not for their specificity regarding a certain subpopulation of differentiated cells within the intestinal epithelia, but for their widespread expression within cycling cells in the crypt for CD44 (e.g., Lgr5+ stem cells and transit amplifying cells) (Wang et al, 2013) and in differentiated enterocytes for AldoB (Beumer et al, 2022; Cosovanu et al, 2022). For examining distinct cell populations, methods such as single-cell RNA sequencing or FACS sorting are better suited (Yang et al, 2025). However, these methodologies require a higher number of organoids, making it impractical to systematically test multiple conditions within an experimental setup. We acknowledge that our methodology is limited in identifying the multiple cell populations that make up the complex intestinal epithelium; nevertheless, it allows us to examine multiple growth conditions, seeded in individual culture wells, and obtain measurements from several replicate experiments.

**Figure 7.**
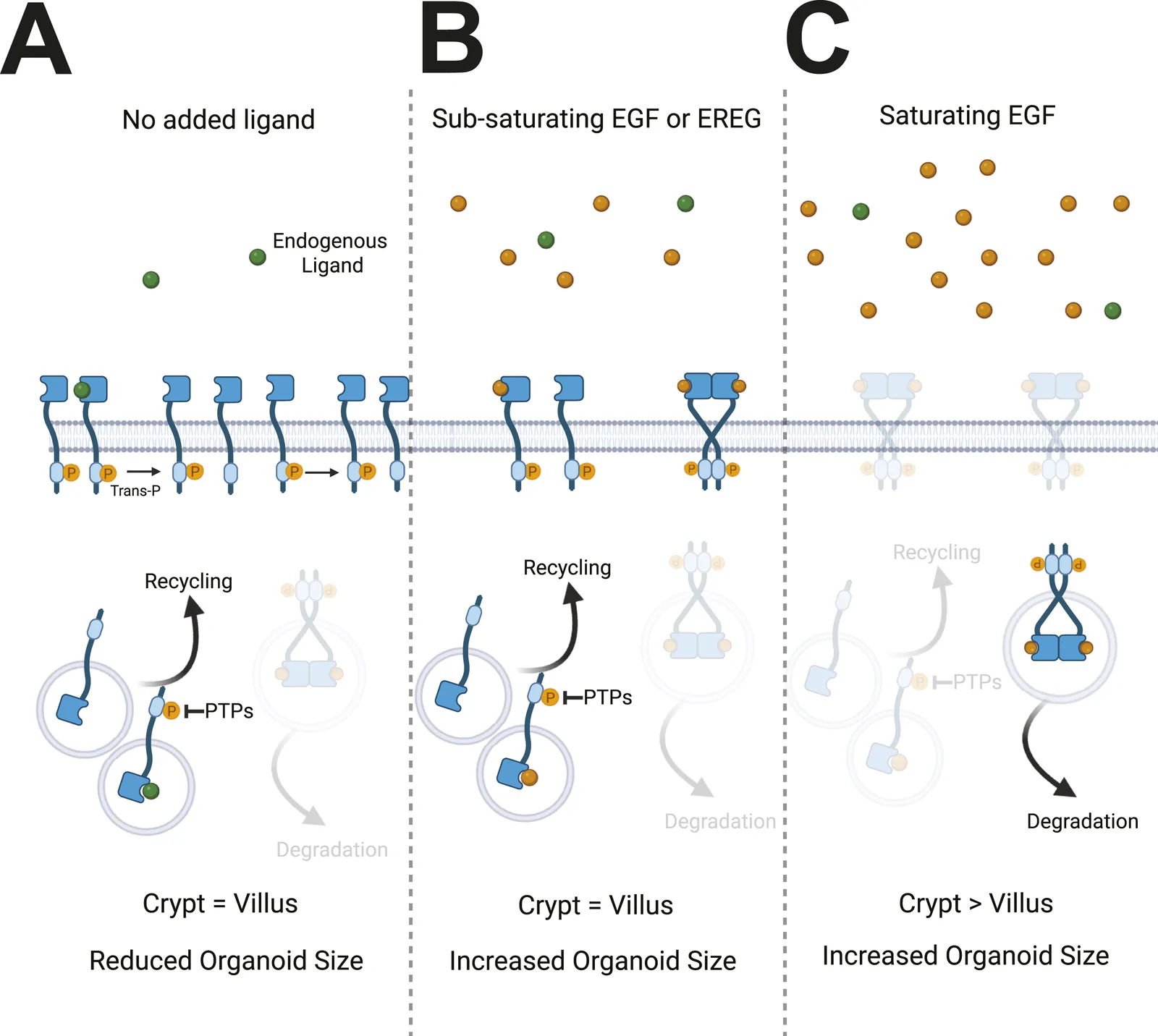
Model and hypothesis for the role of PM localized EGFR in organoid development. **(A)** No exogenous ligand added allows for higher EGFR PM localization and enhanced trans-phosphorylation (Trans-P) of EGFR monomers, likely in response to endogenously expressed ligands. Ligand-less receptors are constantly being recycled back to the PM and protein tyrosine phosphatases (PTPs) are available to remove phosphorylation. In this state, organoids are reduced in size but exhibit equal development of both the crypt and villus regions. **(B)** Use of sub-saturating amounts of EGF (below the normal 50 ng/ml) and low-affinity ligands such as EREG reduce EGFR expression at the PM, thereby reducing total receptor levels and phosphorylation. Under these conditions, receptor recycling to the PM is favored over receptor degradation, leading to even development of the crypt and villus regions and increased overall organoid size. **(C)** Finally, saturating amounts of EGF promote EGFR receptor dimerization and target them for degradation by late endosomes, reducing PM and total EGFR levels. This treatment, normally used in multiple organoid systems, promotes crypt proliferation over villus differentiation. Scheme was created with BioRender.com.

Regardless of which ligands are present during development, they will differentially interact with EGFR in the PM, inducing internalization and trafficking, either promoting receptor recycling back to the PM or degradation within late endosomes (Fig 7). Based on our results, we hypothesize that saturating amounts of EGF commonly used in organoid models target EGFR for late-endosomal degradation, thereby greatly reducing PM receptor levels and promoting a proliferative state over a differentiation program (Fig 7C). At the opposite side of ligand availability, growing organoids without added EGF-family ligands (Fig 7A) led to smaller organoids; however, while NR-grown organoids often lack protruding crypt regions, many times bulging regions are CD44+ and are spatially separated from an ALDO+ region, suggesting that overall development is not affected and is likely maintained by endogenous production of ligands. Furthermore, the high density of PM-located EGFR receptors could in turn make the receptor more susceptible to phosphorylation and trans-phosphorylation of EGFR monomers (Baumdick et al, 2015). Even when unliganded, EGFR receptors are constantly internalized and recycled back to the PM, and in the endosomal compartment, phosphorylated receptors are also targets for protein tyrosine phosphatases, thereby suppressing receptor signaling (Fig 7A). Finally, at low EGF ligand availability or low-affinity ligand presence such as EREG, PM EGFR is increased relative to saturating EGF, leading to a similar organoid size. However, while saturating EGF differentially enhanced crypt development, this condition had a more balanced effect, enhancing both crypt and villus development similarly (Fig 7B).

This hypothesis relies on a wealth of knowledge generated in cell culture monolayers, and the extent to which these endosomal and signaling dynamics ultimately affect individual cellular populations within a mouse intestinal epithelium is currently unknown. EGF-647 endocytosis showed that EGF-647 is primarily internalized in the Ki67-or CD44-labeled crypt area. Labeled EGF has been extensively used to quantify surface levels and/or total receptor levels in cell lines (Roepstorff et al, 2009; Stanoev et al, 2018; Joshi et al, 2023). This process usually includes incubations and washes with ice-cold buffers. These treatments are not possible in organoids, where cold washes could compromise the integrity of the extracellular matrix (Matrigel) that sustains organoid morphology and the intracellular cytoskeletal network. In all conditions tested, we observe less endocytosis in organoids grown with saturating EGF; however, we cannot discard the possibility that EGF-647 could be internalized through smaller vesicles not detectable by CLSM, as EGF endocytosis uses both clathrin- and non-clathrin-mediated endocytic paths, resulting in distinct vesicle populations (Koseska & Bastiaens, 2020).

Ligand removal and introduction experiments highlighted the robustness of EGFR-associated signaling, as ligand signaling inputs introduced at different stages of organoid development could induce convergent phenotypic responses at the end of the 96 h developmental window. The more surprising result showed that 1 h of EGF incubation of freshly seeded single crypts was sufficient to induce organoid development comparable to that induced by 96 h of constant EGF incubation. Experiments in cell culture monolayers often show that brief incubations with EGF, in the range of seconds to minutes, are sufficient to trigger downstream signaling activation as measured in Western blots (Baumdick et al, 2015; Stanoev et al, 2018) or more sensitive methods such as FRET (Ponsioen et al, 2021; Weisheng et al, 2025). In contrast, how these “brief” signaling inputs ultimately affect biological systems in the long term (hours to days after signaling input) is not commonly examined, despite multiple examples in embryonic development (Sonnen & Janda, 2021). Based on our results, we can hypothesize that 1 h incubation with saturating EGF concentration was sufficient to initially deplete EGFR PM membrane levels and then allow for 95 h of EGFR replenishment. The presence of EGFR at the PM led to greater villus differentiation, resembling treatments with EREG or sub-saturating EGF concentrations.

At present, we are unable to determine how treatments that allow EGFR receptor presence in the PM, via endocytic recycling, lead to differential organoid development compared with NR or saturating EGF treatments. It is likely that EGFR in the PM makes organoids more susceptible to fluctuations in endogenously expressed ligand concentrations, thereby modifying organoid proliferation and differentiation patterns. In addition, how endosomal signaling ultimately differs from PM signaling is currently a controversial topic, as downstream effectors remain associated with EGFR long after PM endocytosis (Lin et al, 2026). We showed that EGF and EREG treatments greatly reduced total EGFR levels as shown by immunoblot and IF experiments; the actual role of downstream signaling from phosphorylated EGFR could not be properly assessed in this work. As EGF and EREG are associated with transient and sustained signaling responses (Freed et al, 2017), respectively, and because endocytic cycling facilitates interactions with PTPs, regulation of EGFR phosphorylation, whether at the PM or endosomes, could play a substantial role in the phenotypic outputs we observe.

## Materials and Methods

### Mouse intestinal organoids culture

AdDF+++ medium containing Advanced Dulbecco’s Modified Eagle’s Medium/F12 supplemented with 100 μ/ml penicillin/streptomycin, 10 mM HEPES, 2 mM Glutamax. ENR medium consists of AdDF+++ supplemented with 1x B27 (Life Technologies), 1.25 mM N-acetylcysteine, 50 ng/ml mEGF (315-09; Peprotech), 500 ng/ml R-Spondin 1 (315-32; Peprotech), and 100 ng/ml Noggin (250-38; Peprotech). Alternatively, medium without EGF was used as “NR medium.” Cryopreserved mouse small intestinal organoids were purchased from STEMCELL Technologies (Cat #70931) and grown in Matrigel (354230; Corning Matrigel Growth Factor Reduced [GFR] Basement Membrane Matrix, LDEV-Free) 30 μl droplets in 24-well plates in ENR medium for 96–120 h before splitting by mechanical disintegration using yellow pipette tips. Media was changed every 48 h when single crypts were expanded in EGF-containing media for subsequent experiments. For treatments, NR medium was supplemented with 50 ng/ml of EREG (1195-EP-025; Biotechne) or 50 ng/ml NRG1 (377-HB-050/CF; Biotechne). For media changes, old media was aspirated and replaced with fresh media at established time points in each experiment (e.g., 1, 24, 48 h). Organoids were briefly washed with warm AdDF+++ before adding fresh media if the media had a different composition from the original (e.g., 48 h EGF media followed by 48 h NR media).

### Immuno-staining

Single organoid crypts were seeded in 20 μl Matrigel droplets in x-well culture chambers, 8 wells on coverslips (94.6190.802; Sarstedt). After 96 h of growth, organoids were fixed for 40 min in 4% PFA. Then washed 3 times with IF-buffer (0.2% Triton X100, 0.05% Tween-20) for 5 min, then permeabilized with PBS containing 0.5% Triton X100 for 1 h. Organoids were then blocked with IF-Buffer supplemented with 3% BSA. Alternatively, for staining of phosphorylated EGFR (pEGFR), BSA was replaced by 5% Goat Serum (50197Z; Invitrogen) diluted in IF buffer for both blocking and antibody staining. Primary antibodies CD44 (1:500; 560568; BD Pharmigen), Lysozyme (1:1,000, A0099; DAKO) and Aldolase (1:500, ab153828; Abcam), pEGFR (1:250, EP774Y; Abcam), Ki-67 (1:500, 55060; BD Biosciences), Rab7 (1:500, D95F2; Cell Signaling Technology), Chr-A (1:50, sc-393941; Santa Cruz Biotechnology), DCAMKL1 (1:500, AB109029; Abcam), were incubated overnight with agitation at 4°C. Organoids were washed three times with IF Buffer for 5 min and then incubated for 1 h with Alexa Fluor secondary antibodies (1:500, Thermo Fisher Scientific) and DAPI (1:1,000). Alexa Fluor 488 (A11008, A11001), Alexa Fluor 546 (A11081, A11030), and Alexa Fluor 647 (A31573, A21247) secondary antibodies were used. After washing with IF buffer, samples were kept in Clearing Solution (50% vol/vol Glycerol, 2.5 M Fructose) up to imaging.

### Confocal laser scanning microscopy (CLSM) imaging

Organoids were imaged using Leica Confocal TCS SP8 operated with LASX software (Leica Microsystems) with a 20×/0.75 NA air objective equipped with Laser Kit WLL2 + Pulse Picker and 405 diode laser for illumination. Alternatively, organoid crypts were imaged with 63× 1.4 NA oil-immersion objective for pEGFR, CD44 and EGF647 endocytosis imaging. 2 μm spaced z-stacks were acquired in the fluorescence channels suitable for each sample. Area quantifications were performed using binary masks thresholded from Maximum Intensity Projections of DAPI, CD44, or AldoB, representing the total, crypt, and villus areas, respectively. For Lyz, CHGA or DCAMKL1 imaging, single cells were manually counted from Maximum intensity projections. Image analyses were performed with Fiji/ImageJ software.

### EGF-647 endocytosis

The His-CBD-Intein-(Cys)-hEGF-(Cys) plasmid was kindly provided by Prof. Luc Brunsveld at the University of Technology, Eindhoven. EGF-647 was purified in-house as previously described (Sonntag et al, 2014; Joshi et al, 2023). For endocytosis in organoids, the medium was aspirated from x-well culture chambers (8 wells on coverslips; 94.6190.802; Sarstedt) and rinsed once with warm AdDF+++. Subsequently, organoids were incubated for 30 min with 50 ng/ml of EGF-647 before fixation with 4% PFA. Organoids were imaged on a Leica Confocal SP8 with a 63× oil-immersion objective; crypts were identified by Ki-67 staining. Cellular area was masked with Ki-67 and Rab7 for crypts, and AldoB for villi, and the number of vesicles within the masked regions was quantified using the find maxima command in Fiji.

### Immunoblotting

**Figure S8.**
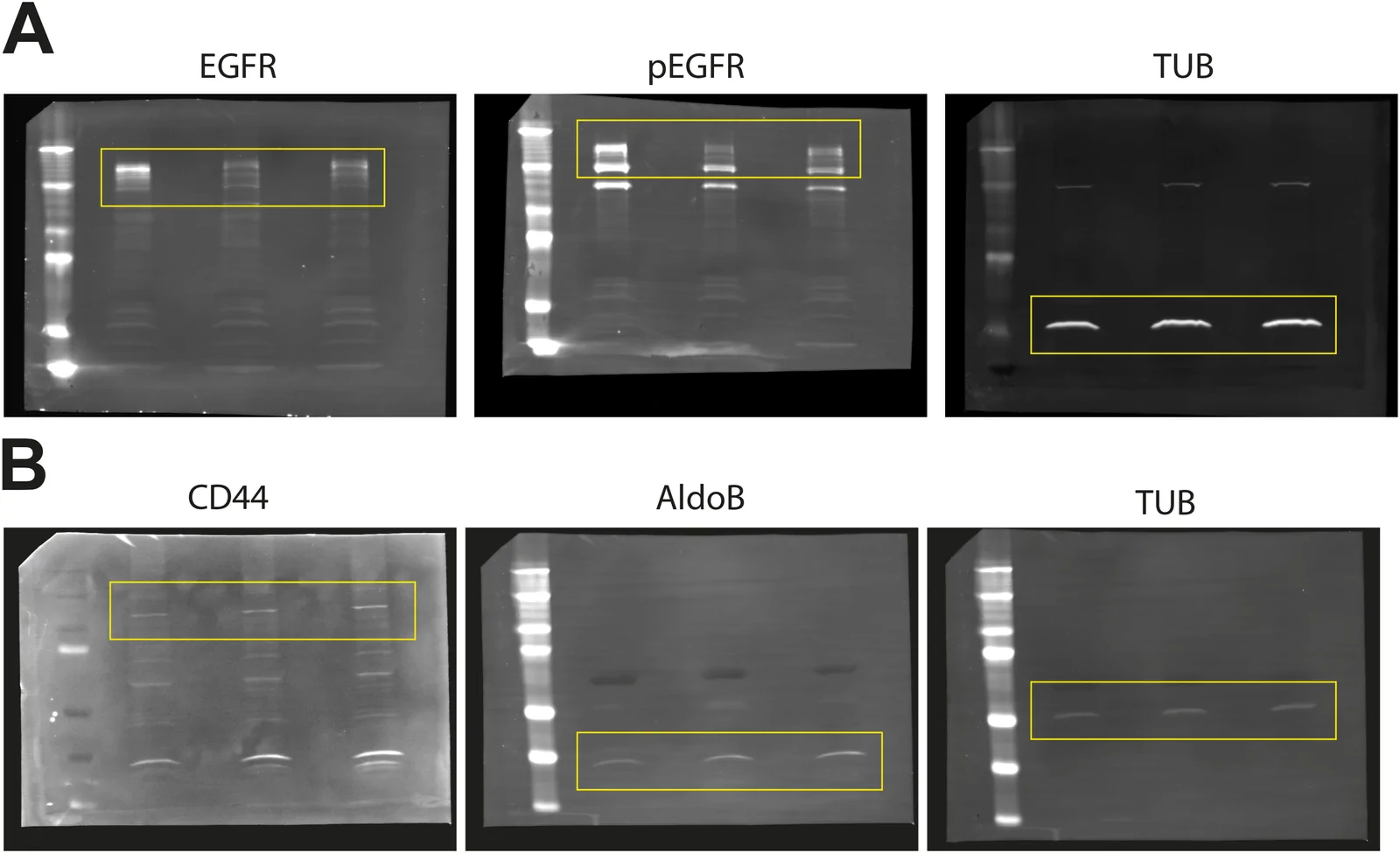
Uncropped immunoblot membranes. **(A)** Full membrane imaging of insets shown in Fig 2C, yellow rectangle denotes cropped region. From left to right, immunoblots from EGFR, pEGFR, and TUB are shown. **(B)** Full membrane imaging of insets shown in Fig S1B, yellow rectangle denotes cropped region. From left to right, immunoblots from CD44, AldoB, and TUB are shown.

Organoid samples were pelleted, resuspended in modified RIPA buffer (50 mM Tris HCl, pH 7.4, 150 mM NaCl, 1 mM EDTA, 1 mM EGTA, 0.5% Triton X-100, and 0.5% sodium deoxycholate) supplemented with Complete Mini EDTA-free protease inhibitor (Roche Applied Science) and phosphatase inhibitor cocktail 2 and 3 (1:100, P5726 and P0044; Sigma-Aldrich), frozen in liquid nitrogen and stored in −70°C freezer. Samples were then dissociated using 10-gauge needle syringes and centrifuged at 15,200*g* for 15 min at 4°C. Protein concentration was determined by BCA (23235; Thermo Fisher Scientific), and 50 g of protein was loaded in 8% SDS/PAGE gels and then transferred onto PVDF membranes. Membranes were then blocked in intercept buffer and incubated with primary antibody overnight. EGFR (C75B9) (1:1,000, 2646; Cell Signaling Technology) for total EGFR, EGFR Y1068 (AP774Y) (1:500; 40815; Abcam) for phospho-EGFR (pEGFR), and α-Tubulin (1:10,000; T6074; Merck) were used. Secondary antibodies IRDye 680 donkey anti-Rabbit IgG (1:10,000), IRDye 800 donkey anti-Mouse IgG (1:10,000) and IRDye 800 donkey anti-Rabbit IgG (1:10,000) (LI-COR Biosciences) were used. Un-cropped full membranes can be found in Fig S8.

### Quantitative RealTime-PCR (qRT-PCR)

mSIOs were grown for 96 h under described experimental conditions and RNA was extracted using RNeasy Mini Kit (74104; Quiagen) according to the manufacturer’s directions. Superscript IV Reverse Trascriptase (Invitrogen) was used for reverse transcription using Oligo dT-primer into cDNA. Triplicates of 25 ml reactions were loaded into 96-well plates and measured on a BioRad CFX96 using PowerUp SYBR Green Master Mix (A25776; Applied Biosystems). *Gapdh* was used as reference gene. 96 h of growth in continuous EGF was used as control. Primers used in this assay are shown in Table 1. Results shown were obtained from N = 3 independent biological replicates. Relative expression was calculated by standard ΔΔC_t_-method including a correction for different cDNA-dilutions for target and reference genes. Different cDNA dilutions were used to compare highly expressed reference genes with low expressed target genes, as for example a 10x higher dilution of cDNA for the qRT-PCR of reference genes. The calculated 2^−ΔΔ^^Ct^ represents the expression foldchange of the target mRNA between sample and EGF 96 h control.

$$corrected\;C_{t}\left(corr.\;C_{t}\right)=C_{t}-\log_{2}dilution\;factor$$

$$\Delta C_{t}=corr.\;C_{t}\left(Target\;Gene\right)-corr.\;C_{t}\left(Reference\;Gene\right)$$

$$\Delta \Delta C_{t}=\Delta C_{t}\left(Sample\right)-Average\Delta C_{t}\left(Control\right)$$

**Table 1.** Primers for qRT-PCR.

​	Forward	Reverse
*AldoB*	GCT​GGG​CAA​TTT​CAG​AGA​GC	GAG​GAC​TCT​TCC​CCT​TTG​CT
*Cd44*	AGC​AGC​GGC​TCC​ACC​ATC​GAG​A	TCG​GAT​CCA​TGA​GTC​ACA​GTG
*Gapdh*	CTC​CCA​CTC​TTC​CAC​CTT​CG	GCC​TCT​CTT​GCT​AGT​GTC​C
*Areg*	CTC​CAC​AGG​GGA​CTA​CGA​CTA	GGG​CTT​AAT​CAC​CTG​TTC​AAC​T
*Egf*	TGA​TGG​GAA​ACA​ATG​TCA​CG	ACC​TGC​AGG​ACA​GAT​GCA​C
*Hbegf*	GAG​TTC​CGT​ACT​CCC​TCT​TGC​A	CAG​CCA​AGA​CTG​TAG​TGT​GGT​C
*Tgfa*	CAG​GCT​CTG​GAG​AAC​AGC​ACA​T	GAC​ACA​TGC​TGG​CTT​CTC​TTC​C

### Digital lightsheet (DLS) microscopy

Organoids were grown in U-Shaped Capillaries 20 × 1.5 mm (158007061; Leica) inside Glass-Bottom 35 mm MatTek Dishes (P35G-1.5-10-C; MatTek Life Sciences) for 96 h in NR medium. Before imaging, organoids were treated for 1 h with 1:500 Cell Mask Green Actin Tracking stain (A57243; Invitrogen) and Hoechst 33342 (C10340 G; Invitrogen). Afterward organoids were washed twice with warm AdDF+++ medium and returned to fresh NR medium. Organoids were imaged on a Leica TCS SP8 operated with LASX software (Leica Microsystems) Digital Lightsheet (DLS) DMi850 using an HC PL FLUOTAR 5×/0.15 excitation objective and a FLUOTAR L25×/0.95 W DLS detection objective. Laser Kit WLL2 + Pulse Picker and 405 diode laser were used. 50 ng/ml of EGF-647 was added after calibration and ROI selection.

### Statistical analyses

Experiments for Figs 1, 2, 4, and 6 were performed in parallel; results are shown in separate figures and graphs for clarity. As experimental settings are compared with 96-h treatments with NR, EGF, or EREG, the corresponding control quantifications are repeated in the scatter plots. Experiments for Fig 5 were performed independently and at a separate time. Experiments for Fig S6 were also performed at the same time; thus, 96-h controls are repeated among the scatter plots.

Analysis was performed with at least three biological replicates (N = 3) unless otherwise stated. Scatter plots show mean ± 95% CI and bar graphs show mean ± SD. For single group comparisons Krustal-Wallis with Dunn’s multiple comparisons test was used. For multiple group comparisons Two-way ANOVA with Sidak’s multiple comparisons test was used. *P*-values <0.05 are shown. All analyses and graphs were performed using GraphPad Prism10 V106.1.

## Supplementary Material

Reviewer comments

## Data Availability

Data available upon reasonable request.
