# Supplementary material for: Proliferation and differentiation in intestinal organoids are balanced by ligand-modulated EGFR trafficking
Source: Life Sci Alliance. 2026 Aug 27;9(11):e202603733. doi: 10.26508/lsa.202603733 (PMC13524303; doi:10.26508/lsa.202603733)
Supplement: Supplementary file 1 [file LSA-2026-03733_review_history.pdf]

Proliferation and differentiation in intestinal organoids are balanced by ligand-modulated EGFR trafficking

Mario O. Caracci, Sabrina Seidler, Luis M. Munoz-Nava, Birga Soetje, Kirsten Michel and Philippe I.H. Bastiaens
DOI: https://doi.org/10.26508/lsa.202603733

Corresponding author(s): Dr. Mario O. Caracci (Max Planck Institute of Molecular Physiology)

Review Timeline:

Submission Date:	2026-04-08
Editorial Decision:	2026-05-15
Revision Received:	2026-07-12
Editorial Decision:	2026-08-19
Revision Received:	2026-08-20
Accepted:	2026-08-21

Scientific Editor: Tim Fessenden

Transaction Report:

(Note: With the exception of the correction of typographical or spelling errors that could be a source of ambiguity, letters and reports are not edited. The original formatting of letters and referee reports may not be reflected in this compilation.)

May 15, 2026

Re: Life Science Alliance manuscript #LSA-2026-03733-T

Dr. Mario O. Caracci
Max Planck Institute of Molecular Physiology
Systemic Cell Biology
Otto-Hahn-Straße 11
Dortmund, Nordrhein Westfalen 44227
Germany

Dear Dr. Caracci,

Thank you for submitting your manuscript entitled "Proliferation/Differentiation in intestinal organoids as a balance of ligand-modulated EGFR traffic" to Life Science Alliance. The manuscript was assessed by expert reviewers, whose comments are appended to this letter.

As you will see, all reviewers remarked on the value of this systematic assessment of gut organoid proliferation and differentiation regulated by EGFR ligand identity, concentration, and duration. However all reviewers pointed to concerns that limit the strength of the conclusions and the clarity of the results presented. We invite you to submit a revised manuscript addressing their main concerns.

Namely, Reviewer 1 noted that auto/paracrine signaling of EGF ligands within organoids may significantly impact these observations, and we concur that their points 1 and 2 must be addressed. Per Reviewer 2, crypt/villus markers used for growth measurements should be validated with an orthogonal method of your choice, and if possible a broader accounting of differentiated cell types in organoids sought by Reviewer 3 would be valuable but remains optional. Finally we agree with Reviewers 2 and 3 that the text must significantly improve for this manuscript to be suitable for publication. We appreciate the attention of Reviewer 3 to the details in the text, although we refer you to LSA's guidance on formatting the reference section.

I would be happy to discuss the revision in more detail via email or phone/videoconferencing. Please let me know which option you prefer, if any.

To upload the revised version of your manuscript, please log in to your account: https://lsa.msubmit.net/cgi-bin/main.plex

You will be guided to complete the submission of your revised manuscript and to fill in all necessary information. Please get in touch in case you do not know or remember your login name.

The typical timeframe for revisions is three months. Please note that papers are generally considered through only one revision cycle, so strong support from the referees on the revised version is needed for acceptance.

While you are revising your manuscript, please also attend to the below editorial points to help expedite the publication of your manuscript. Please direct any editorial questions to the journal office. When submitting the revision, please include a letter addressing the reviewers' comments point by point.

Thank you for this interesting contribution to Life Science Alliance. We hope that the comments below will prove constructive as your work progresses, and we are looking forward to receiving your revised manuscript.

Sincerely,

Tim Fessenden
Scientific Editor
Life Science Alliance

A. THESE ITEMS ARE REQUIRED FOR REVISIONS

-- A letter addressing the reviewers' comments point by point.

-- An editable version of the final text (.DOC or .DOCX) is needed for copyediting (no PDFs).

-- High-resolution figure, supplementary figure and video files uploaded as individual files: See our detailed guidelines for preparing your production-ready images, https://www.life-science-alliance.org/authors

-- Summary blurb (enter in submission system): A short text summarizing in a single sentence the study (max. 200 characters including spaces). This text is used in conjunction with the titles of papers, hence should be informative and complementary to the title and running title. It should describe the context and significance of the findings for a general readership; it should be written in the present tense and refer to the work in the third person. Author names should not be mentioned.

-- By submitting a revision, you attest that you are aware of our payment policies found here: https://www.life-science-alliance.org/copyright-license-fee

B. MANUSCRIPT ORGANIZATION AND FORMATTING:

Full guidelines are available on our Instructions for Authors page, https://www.life-science-alliance.org/authors

We encourage our authors to provide original source data, particularly uncropped/-processed electrophoretic blots and spreadsheets for the main figures of the manuscript. If you would like to add source data, we would welcome one PDF/Excel-file per figure for this information. These files will be linked as supplementary "Source Data" files.

IMPORTANT: It is Life Science Alliance policy that if requested, original data images must be made available. Failure to provide original images upon request will result in unavoidable delays in publication. Please ensure that you have access to all original microscopy and blot data images before submitting your revision.

Reviewer #1 (Comments to the Authors (Required)):

The manuscript titled 'Proliferation/Differentiation in intestinal organoids as a balance of ligand-modulated EGFR traffic', by Caracci and Bastiaens and colleagues, explores how distinct EGFR ligands differentially regulate the growth and development of intestinal organoid cultures. This question has been previously addressed by other studies, and has important implications for both a basic understanding of EGFR signaling in biology, as well as for translational efforts to prevent and/or treat inflammatory bowel disease and colorectal cancer. The current study attempts to address this question in more depth than prior studies, and thus presents some value to this important emerging field. However, several major limitations exist that must be addressed:

Major concerns:

1.) Regarding the consideration of organoid-intrinsic EGFR ligand production: the authors acknowledge that Paneth cells are reported to produce EGF. Any organoid-intrinsic production of EGFR ligands severely limits the interpretation of experiments where exogenous EGFR ligand is added. The authors must address this caveat in the interpretation of each experiment.

2.) In addition to Paneth cell production of EGF, many public scRNAseq datasets show EGFR ligand expression (notably Ereg and Areg) within intestinal epithelial cells. The authors should carefully assess the expression of organoid-intrinsic EGFR ligand expression, for all seven EGFR ligands, in all the ligand addition formats tested here. Since good antibodies may not be available, PCR-based analysis of mRNA would be sufficient. These results then must be considered in the interpretation of any data. For example, if organoid-intrinsic expression of any EGFR ligand is increased or decreased depending on exogenous ligand stimulations conditions, then that adds significant caveats to interpretation of the data.

3.) Because organoids contain diverse epithelial cell subsets, the authors can not make any strong conclusions regarding the relative role of EGFR signaling within any individual cell type. For example, it remains unclear whether differences in villus formation under different ligand conditions owe to differences in EGFR signaling within stem cells, or within transit-amplifying cells. This is a major limitation of the study, which must be addressed directly in the interpetation.

Minor concerns:
1.) In the concluding remarks, the authors state that EGF expression is upregulated in Paneth cells after tissue injury. The authors fail to cite or discuss the extensive literature that demonstrate a role for Ereg and Areg most notably, along with TGFalpha and HB-EGF, in ameliorating intestinal tissue damage. The authors should cite and briefly discuss this literature.

Reviewer #2 (Comments to the Authors (Required)):

The manuscript by Caracci et al: "Proliferation/Differentiation in intestinal organoids as a balance of ligand-modulated EGFR traffic" investigates the impact of EGFR ligands EGF and EREG in the growth of mouse intestinal organoids. They compartmentalize the organoids to villus and crypt subsets and investigate the effects of different ligands, their dosage timing,

and concentration, to villus and crypt growth as well as receptor internalization. The study contains interesting elements, and the segmentation of organoid growth to crypt proliferation and differentiation by measuring different parts of the organoid is innovative. Also imaging endocytosis in intestinal organoids is an interesting new approach.
Concerns to be addressed:
1. It is interesting that the organoids appear to grow well without EGF (NR conditions). This is not usually seen in the literature despite the knowledge that Paneth cells secrete EGF ligands. Please elaborate.
2. The imaging of crypts and villus parts separately is interesting and a key advance in this manuscript. An independent method (e.g. Western blot of AldoB and CD44 or quantitative qPCR using crypt/villus specific genes) to validate the ratio of changing the crypt/villus ratio would strengthen the interpretation.
3. Fig1C. What do numbers 1, 48 and 96 indicate in this experiment?
4. In Figure 2A, it's interesting that 1 hour of treatment with EGF is sufficient to maintain the organoid growth. Is it possible to separate the AldoB+ area from the autofluorescence of the dead cells inside the "villus" region of the crypt?
5. Fig 1F and Fig2H NR condition: the plots looks identical. Are these separate experiments or was NR data brought from a different experiment?
6. The discussion appears not finalized. E.g. the paragraph discussing the clathrin-mediated endocytosis should be rewritten to clarify what is being said.
7. A schematic picture of the findings and the author's interpretation on timing, dosing, and EGFR ligand-specificity to crypt/villus growth would be helpful.
8. There are spelling mistakes throughout the manuscript and parts of the materials and methods appear preliminary (e.g. "single organoid crypts (row 391), X-well). Was 50% Goat serum really used? Row 423 should be revised.

Reviewer #3 (Comments to the Authors (Required)):

The manuscript submitted by the authors details their investigations into how EGFR ligands present in the media of in vitro grown small intestinal organoids affect their differentiation and proliferation. The manuscript definitely presents some necessary information related to how small intestinal organoids are grown and how the presence of specific types and amounts of EGFR ligands affect their growth and differentiation. However, the manuscript is difficult to understand in parts with many grammatical mistakes. Most concerning is the significant lack of figure references. There are a multitude of areas in the manuscript where conclusions and results are described but no reference to a specific figure (or reference to literature) is listed. This makes the manuscript difficult to understand and seeds a concerning level of doubt in the findings that are present. Detailed below are some of the major concerns I have with the manuscripts and what might be done to fix it. I strongly support this type of work as it is critical to have a good understanding of the media and ligands used to grow organoids if we are to use this tool as a means to better understand crypt growth. Therefore, I feel as though this manuscript could be considered for publication but would have to be heavily modified and then re-evaluated again before a decision can be made.
Major concerns:
EGF and EREG mediated endocytosis reduce EGFR PM localization and expression:
• The sentence on lines 145-147 states that most of the internalized EGF-647 is located in the crypt regains with few being observed in the villus regions. There is no figure reference to support this claim. The authors would need to provide data showing EGFR-647 internalization relative to CD44 vs AldoB immunofluorescence with quantification to say this.
• The result presented in line 148-150 is intriguing, however, the reason for the lack of EGF-647 internalization is likely due to saturating levels of EGF present in the media. This conclusion is hinted at later in the manuscript, but it would be good to put it here first.
• The sentence on line 173-176 is very confusing as it is written. The results clearly show decreased pEGFR in organoids grown in EGF or EREG, however, how CD44 fits into this is not adequately explained and does not due the data justice. It would be good to take a couple of sentences to describe the results a bit better and with more detail.
• The very last paragraph has some very important conclusions related to EGF secretion by Paneth cells and its effect on EGFR phosphorylation. However, as it is written, it is not clear how the bit related to media changes and pulses of EGF and EREG are relevant to the overall point of this section. This confusion is made worse by last sentence, which is especially confusing. As it is written, it doesn't fit with what the data shows.
• It would be of use to also characterize what differentiated cell types are present in the various media conditions. This can be accomplished by immunofluorescence and would increase the support that the organoids are indeed differentiated. As an extension of this, it would also be good to examine markers of the intestinal stem cell population that should be present in the CD44+ crypt area of the organoids.
Temporal modulation of EGF-family ligands during development alters villus differentiation.
• This section was weakest out of the whole manuscript. It was very difficult to even evaluate due to the significant lack of figure references. There were too many instances to exhaustively list here in which a result was described, but no figure was referenced. It can't be stressed enough that without a reference to a figure (even if it is repetitive) helps the reader stay on track with author's description of the results.
• A better description of how the organoids were split and when the media was added is also necessary here. Are these media conditions present right at splitting? Is the media change happening only one hour after the organoids are seeded? Or are the media changes occurring in organoids that are already established. A diagram detailing when the organoids are split and when the various media changes happen is necessary to be able to evaluate this section.

• The diagram present in Figure 2A indicates that EGF is added for the first hour and then it is removed and replaced with NR media. One hour doesn't seem a long enough period to result in any effect whatsoever on organoid growth.
• I can't stress enough that there is likely good data in this section. However, without a proper explanation of when media changes are occurring in relation to seeding the organoids a proper evaluation of the experiments is not possible. This section would need to be rewritten, adding in a better description of the methods with a better diagram and then re-evaluated before conclusions can be made related to the veracity of the results and any additional experiments needed.
Sub-saturating concentrations of EGF enhance both crypt proliferation and villus differentiation:
• As seen in the last sections, this section lacked a few instances where a refecen to a figure is needed. For example, the sentence on line 282-283 clearly reference data but has no figure reference at the end. The same goes for lines 288-289.
• As stated above, a better characterization of crypt and villus differentiation beyond CD44 and AldoB is needed to evaluate how well the organoids are differentiating.
Multiple pulses of EGF deplete EGFR surface expression reducing villus differentiation:
• Line 306 to 308 lacks reference to a specific figure.
Minor concerns:
• In vitro should always be written in italics.
• The references do not list all of the authors, but instead seem to only list a certain number of authors with no et al., after the last author. In the reference section, all authors associated with a given publication should be listed.
• There are a number of spots where conclusions are stated but no reference is listed. Below are two examples, but this is by no means an exhaustive list. Authors should always list a reference that was used to make a conclusion.
o The sentence ending on line 52 needs a reference so the reader can find the study that led the authors to reach this conclusion.
o The same goes for the sentence ending on line 61.
• The entire manuscript should be reviewed for grammatical mistakes; there were too many to provide an exhaustive list.
• Abbreviations used in the manuscript should be spelt out first with the abbreviation in parentheses. All references after this can be made using the abbreviation.
• The graphs in figure 2 should start with the data for 1 hour and then proceed up to 96.

May 15, 2026

Re: Life Science Alliance manuscript #LSA-2026-03733-T

Dr. Mario O. Caracci
Max Planck Institute of Molecular Physiology
Systemic Cell Biology
Otto-Hahn-Straße 11
Dortmund, Nordrhein Westfalen 44227
Germany

Dear Dr. Caracci,

Thank you for submitting your manuscript entitled "Proliferation/Differentiation in intestinal organoids as a balance of ligand-modulated EGFR traffic" to Life Science Alliance. The manuscript was assessed by expert reviewers, whose comments are appended to this letter.

As you will see, all reviewers remarked on the value of this systematic assessment of gut organoid proliferation and differentiation regulated by EGFR ligand identity, concentration, and duration. However all reviewers pointed to concerns that limit the strength of the conclusions and the clarity of the results presented. We invite you to submit a revised manuscript addressing their main concerns.

Namely, Reviewer 1 noted that auto/paracrine signaling of EGF ligands within organoids may significantly impact these observations, and we concur that their points 1 and 2 must be addressed. Per Reviewer 2, crypt/villus markers used for growth measurements should be validated with an orthogonal method of your choice, and if possible a broader accounting of differentiated cell types in organoids sought by Reviewer 3 would be valuable but remains optional. Finally we agree with Reviewers 2 and 3 that the text must significantly improve for this manuscript to be suitable for publication. We appreciate the attention of Reviewer 3 to the details in the text, although we refer you to LSA's guidance on formatting the reference section.

I would be happy to discuss the revision in more detail via email or phone/videoconferencing. Please let me know which option you prefer, if any.

To upload the revised version of your manuscript, please log in to your account: https://lsa.msubmit.net/cgi-bin/main.plex

You will be guided to complete the submission of your revised manuscript and to fill in all necessary information. Please get in touch in case you do not know or remember your login name.

The typical timeframe for revisions is three months. Please note that papers are generally considered through only one revision cycle, so strong support from the referees on the revised version is needed for acceptance.

While you are revising your manuscript, please also attend to the below editorial points to help expedite the publication of your manuscript. Please direct any editorial questions to the journal office. When submitting the revision, please include a letter addressing the reviewers' comments point by point.

Thank you for this interesting contribution to Life Science Alliance. We hope that the comments below will prove constructive as your work progresses, and we are looking forward to receiving your revised manuscript.

Sincerely,

Tim Fessenden
Scientific Editor
Life Science Alliance

A. THESE ITEMS ARE REQUIRED FOR REVISIONS

-- A letter addressing the reviewers' comments point by point.

-- An editable version of the final text (.DOC or .DOCX) is needed for copyediting (no PDFs).

-- High-resolution figure, supplementary figure and video files uploaded as individual files: See our detailed guidelines for preparing your production-ready images, https://www.life-science-alliance.org/authors

-- Summary blurb (enter in submission system): A short text summarizing in a single sentence the study (max. 200 characters including spaces). This text is used in conjunction with the titles of papers, hence should be informative and complementary to the title and running title. It should describe the context and significance of the findings for a general readership; it should be written in the present tense and refer to the work in the third person. Author names should not be mentioned.

-- By submitting a revision, you attest that you are aware of our payment policies found here: https://www.life-science-alliance.org/copyright-license-fee

B. MANUSCRIPT ORGANIZATION AND FORMATTING:

Full guidelines are available on our Instructions for Authors page, https://www.life-science-alliance.org/authors

We encourage our authors to provide original source data, particularly uncropped/-processed electrophoretic blots and spreadsheets for the main figures of the manuscript. If you would like to add source data, we would welcome one PDF/Excel-file per figure for this information. These files will be linked as supplementary "Source Data" files.

IMPORTANT: It is Life Science Alliance policy that if requested, original data images must be made available. Failure to provide original images upon request will result in unavoidable delays in publication. Please ensure that you have access to all original microscopy and blot data images before submitting your revision.

Reviewer #1 (Comments to the Authors (Required)):

The manuscript titled 'Proliferation/Differentiation in intestinal organoids as a balance of ligand-modulated EGFR traffic', by Caracci and Bastiaens and colleagues, explores how distinct EGFR ligands differentially regulate the growth and development of intestinal organoid cultures. This question has been previously addressed by other studies, and has important implications for both a basic understanding of EGFR signaling in biology, as well as for translational efforts to prevent and/or treat inflammatory bowel disease and colorectal cancer. The current study attempts to address this question in more depth than prior studies, and thus presents some value to this important emerging field. However, several major limitations exist that must be addressed:

We wish to thank the reviewer for his/her helpful comments and interest in our research.

Major concerns:

1.) Regarding the consideration of organoid-intrinsic EGFR ligand production: the authors acknowledge that Paneth cells are reported to produce EGF. Any organoid-intrinsic production of EGFR ligands severely limits the interpretation of experiments where exogenous EGFR ligand is added. The authors must address this caveat in the interpretation of each experiment.

2.) In addition to Paneth cell production of EGF, many public scRNAseq datasets show EGFR ligand expression (notably Ereg and Areg) within intestinal epithelial cells. The authors should carefully assess the expression of organoid-intrinsic EGFR ligand expression, for all seven EGFR ligands, in all the ligand addition formats tested here. Since good antibodies may not be available, PCR-based analysis of mRNA would be sufficient. These results then must be considered in the interpretation of any data. For example, if organoid-intrinsic expression of any EGFR ligand is increased or decreased depending on exogenous ligand stimulations conditions, then that adds significant caveats to interpretation of the data.

It is certainly likely that absence of EGFR ligands in the culture media might induce endogenous expression of other ligands by the intestinal epithelia. As the reviewer points out, in addition to EGF, other ligands such as EREG, AREG, Tgfa, and HbEGF are also expressed by epithelia, according to the literature. However, it is important to note that ligand expression is typically observed in response to tissue damage or inflammation; furthermore, in many cases, the ligands are secreted by stromal cells rather than the epithelia. Many of these ligands have also been shown to promote

mSIOs growth, although at high, non-physiological concentrations (50 ng/ml and above). While tissue geometry might increase ligand concentration, it is unlikely that a high, receptor-saturating amount of ligand would be observed in vivo.

We believe the main finding of this article is how the plasma membrane localization of EGFR is modified by ligands, regardless of the ligand used, triggering differential development of a proliferating crypt region vs a differentiated villus region. As an extension of this conclusion, even if endogenous EGFR ligands are upregulated by any of our treatments, the absence of the EGFR receptor in the plasma membrane, due to excess amounts of exogenous ligands (Any ligand), would render organoids virtually "Blind" to these signals. Signaling competency of EGFR at the plasma membrane could have profound implications in how organoid technology is currently used for disease modeling, as most organoid systems use high concentrations of EGF in their culture media. This is something we wish to explore further in a future publication.

We can modulate membrane EGFR localization by using decreasing amounts of ligands, lower-affinity ligands, or ligand starvation, and observe developmental outcomes consistent with these membrane localization levels. This observation led us to believe that endogenously expressed ligands do not play an active role in triggering proliferation and/or differentiation in mSIOs, although they might play a role in other processes, such as stem cell maintenance.

Since qRT-PCR requires a high number of organoids, it is impractical for us to test this in all the treatment conditions presented in this work (20 independent conditions). We tested ligand expression by qRT-PCR under the treatment conditions depicted in Figure 5, as these experiments capture the main array of developmental changes observed in this work. EGF, AREG, HbEGF, and Tgfa were quantified and showed increased expression in NR medium and at 0.05 ng/mL EGF; no effect was observed with EREG or at higher EGF concentrations. We were unable to obtain amplification of EREG; at this point, we cannot tell if this is due to low expression or technical issues. The literature suggests that EREG is expressed in epithelia; however, the expression is higher upon tissue damage.

We have largely modified the manuscript to comply with Life Science Alliance formatting rules and have added several statements addressing the potential role of endogenously expressed ligands in organoid development:

Sentence 334-344
"Limited availability of extracellular ligands may enhance the endogenous expression of other EGF-family ligands to maintain downstream signaling; furthermore, high and low doses of EGF have been shown to activate distinct transcriptional programs (Weisheng et al., 2025). RT-qPCR showed that while Cd44 and AldoB expression is mostly unchanged (Figure S7A), EGFR ligands such as Egf, Hbegf, Tgfa, and Areg are differentially increased in NR media or in the presence of 0.05 ng/ml EGF (Figure S7B). Expression of these ligands may allow for basal EGFR activation to be maintained in the absence of media-supplemented EGF family ligands. Nevertheless, it does not seem that expression of these ligands ultimately modifies gross development of the crypt and villus regions and very likely are necessary to maintain the stem cell niche in absence of other ligands (Abud et al., 2021)."

Sentence 416-421

"At the opposite side of ligand availability, growing organoids without added EGF-family ligands (Figure 7A) led to smaller organoids; however, while NR-grown organoids often lack protruding crypt regions, many times bulging regions are CD44+ and are spatially separated from an ALDO+ region, suggesting that overall development is not affected and is likely maintained by endogenous production of ligands"

3.) Because organoids contain diverse epithelial cell subsets, the authors can not make any strong conclusions regarding the relative role of EGFR signaling within any individual cell type. For example, it remains unclear whether differences in villus formation under different ligand conditions owe to differences in EGFR signaling within stem cells, or within transit-amplifying cells. This is a major limitation of the study, which must be addressed directly in the interpetation.

In the current version of the manuscript, we have expanded the cell populations examined by adding enteroendocrine cell staining in all our experimental conditions; these results are shown in FigureS6. With that said, we acknowledge that this fact is a major limitation in our work, and we have added the following statement in the text:

Sentence 390-408

"In the present work, we attempt to bridge the vast knowledge currently available on the role of EGF-family ligands in intestinal development with the dynamics of EGFR signaling and the endocytic cycle (Koseska & Bastiaens, 2020; Jendrisek et al, 2026). To test this, we systematically added and removed EGF and EREG, quantified EGFR levels at the PM after treatments, and ultimately assessed the phenotypic effects on organoid development. CD44 and AldoB were used as markers for the proliferative crypt region and the differentiated villus region in mSIOs. We chose these markers not for their specificity regarding a certain subpopulation of differentiated cells within the intestinal epithelia, but for their widespread expression within cycling cells in the crypt for CD44 (eg. Lgr5+ stem cells and transit amplifying cells) (Wang et al, 2013) and for differentiated enterocytes for AldoB (Beumer et al, 2022; Cosovanu et al, 2022). For examining distinct cell populations, methods such as single-cell RNA sequencing or FACS sorting are better suited (Yang et al, 2025). However, these methodologies require a higher number of organoids, making it impractical to systematically test multiple conditions within an experimental setup. We acknowledge that our methodology is limited in identifying the multiple cell populations that make up the complex intestinal epithelium; nevertheless, it allows us to examine multiple growth conditions, seeded in individual culture wells, and obtain measurements from several replicate experiments."

Minor concerns:

1.) In the concluding remarks, the authors state that EGF expression is upregulated in Paneth cells after tissue injury. The authors fail to cite or discuss the extensive literature that demonstrate a role for Ereg and Areg most notably, along with

TGFalpha and HB-EGF, in ameliorating intestinal tissue damage. The authors should cite and briefly discuss this literature.

We have highlighted the importance of other EGF ligands throughout the manuscript:

Sentence 55-59
“Other EGF-family ligands such as Neuregulin (NRG1), Epiregulin (EREG) and Heparin-binding EGF-like growth factor (HB-EGF) can replace EGF in mSIOs culture, promoting organoid development (Lemmetyinen et al., 2023; Jardé et al., 2020; Chen et al., 2012), notably epithelial KO of NRG1 does not affect differentiation of secretory lineage cells (Jardé et al., 2020)”

Sentence 79-85
“For instance, high-affinity ligands such as EGF, HB-EGF, and TGFa, all produced in the intestinal epithelia, are often associated with cell proliferation (Abud et al, 2021; Jendrisek et al, 2026). On the other hand, low-affinity ligands such as EREG and AREG, which are expressed by stromal cells in the intestine, are associated with cell differentiation and migration (Lemmetyinen et al, 2023; Jendrisek et al, 2026)”

Sentence 375-378
“Also, other EGF-family ligands play a substantial role during damage and regeneration processes in the intestine, including HB-EGF and Areg, highlighting the potential role of EGFR-binding ligands during organoid development (Shao & Sheng, 2010; Su et al, 2013).”

Reviewer #2 (Comments to the Authors (Required)):

The manuscript by Caracci et al: "Proliferation/Differentiation in intestinal organoids as a balance of ligand-modulated EGFR traffic" investigates the impact of EGFR ligands EGF and EREG in the growth of mouse intestinal organoids. They compartmentalize the organoids to villus and crypt subsets and investigate the effects of different ligands, their dosage timing, and concentration, to villus and crypt growth as well as receptor internalization. The study contains interesting elements, and the segmentation of organoid growth to crypt proliferation and differentiation by measuring different parts of the organoid is innovative. Also imaging endocytosis in intestinal organoids is an interesting new approach.

We wish to thank the reviewer for his/her kind comments and interest in our research.

Concerns to be addressed:
1. It is interesting that the organoids appear to grow well without EGF (NR conditions). This is not usually seen in the literature despite the knowledge that Paneth cells secrete EGF ligands. Please elaborate.

It is important to note that our experimental setting includes seeding single crypts, not individual stem cells, the presence of a pre-established stem cell niche in this crypts (including Paneth cells) might aid in the early development of this organoids even in absence of exogenous ligands.

Indeed, NR-cultured mSIOs often appear “under-developed”; however, it is not uncommon to find organoids with CD44+ staining confined to small crypts or bulges. NR-grown organoids are, of course, smaller than EGF- or EREG-treated organoids; however, even the smallest organoids show a CD44+ area spatially separated from an AldoB+ area. While we are reluctant to call these CD44+ regions a “crypt”, the distinctive presence of both markers suggests that specification of a crypt and villus region does not necessarily require EGF-ligands; however, efficient proliferation to maintain balanced development does require ligands.

We have added the following paragraph in the discussion section:

Sentence 416-423
“At the opposite side of ligand availability, growing organoids without added EGF-family ligands (Figure 7A) led to smaller organoids; however, while NR-grown organoids often lack protruding crypt regions, many times bulging regions are CD44+ and are spatially separated from an ALDO+ region, suggesting that overall development is not affected and is likely maintained by endogenous production of ligands. Furthermore, the high density of PM-located EGFR receptors could in turn make the receptor more susceptible to phosphorylation and trans-phosphorylation of EGFR monomers (Baumdick et al, 2015).”

2. The imaging of crypts and villus parts separately is interesting and a key advance in this manuscript. An independent method (e.g. Western blot of AldoB and CD44 or quantitative qPCR using crypt/villus specific genes) to validate the ratio of changing the crypt/villus ratio would strengthen the interpretation.

Thank you for your comment. As you can see here, we use both of these markers to separate distinct regions within the organoids that are also observable in the physiological tissue. As such, we do not claim that either of these markers undergoes up or downregulation upon any of the treatments; we just limit their usage to identify regions and quantify them as proof of differential development.

Since qRT-PCR and WB experiments require a high number of organoids, it is impractical for us to test this in all the treatment conditions presented in this work (20 independent conditions). We quantified AldoB and CD44 expression in 96h treatment organoids (Treatments in Figure 1 and control conditions in all other experimental settings), and the results are currently displayed in Figure S1A and B. qRT-PCR and WB showed no differential expression of either marker under the tested conditions. Expression was also quantified by qRT-PCR for the conditions shown in Figure 5.

We have added the following comments in results and discussion:

Sentence 134-138
“Expression levels of CD44 and AldoB did not show any significant changes upon treatments (Figure S1A and B), as these markers have fundamentally a very heterogeneous expression that varies from cell to cell depending on cell age or cell cycle status (Gracz et al, 2013; Zheng et al, 2023).”

Sentence 395-401

“CD44 and AldoB were used as markers for the proliferative crypt region and the differentiated villus region in mSIOs. We chose these markers not for their specificity regarding a certain subpopulation of differentiated cells within the intestinal epithelia, but for their widespread expression within cycling cells in the crypt for CD44 (eg. Lgr5+ stem cells and transit amplifying cells) (Wang et al, 2013) and for differentiated enterocytes for AldoB (Beumer et al, 2022; Cosovanu et al, 2022).”

3. Fig1C. What do numbers 1, 48 and 96 indicate in this experiment?

Those numbers indicate the number of Hours in each specific treatment. “(h)” has been added to all figures. Figure 3,4, S4 and S6B and C

4. In Figure 2A, it's interesting that 1 hour of treatment with EGF is sufficient to maintain the organoid growth. Is it possible to separate the AldoB+ area from the autofluorescence of the dead cells inside the "villus" region of the crypt?

We first want to clarify that the images shown are maximum-intensity projections of multiple confocal slices, so the inner mass of dead cells is not visible.

With that said, within individual confocal slices, we do not observe CD44 or AldoB staining in this inner mass of cells; these antibodies show a very clear signal over background/noise. In some cases, DAPI staining is visible; however, the signal is often lower than that of live cells within the epithelia. Since maximum-intensity projections select the highest-intensity pixel across all slices, the inner cell mass is not visible even in the DAPI channel.

Finally, we have added an experiment (Figure S2) showing a single confocal section labeled with AldoB and Cd44, indicating that the inner cell mass is not observable.

5. Fig 1F and Fig2H NR condition: the plots looks identical. Are these separate experiments or was NR data brought from a different experiment?

Experiments in Figures 1, 2, 4, and 6 were performed in parallel. Given the high number of experimental conditions we used throughout this article (20 independent conditions), we separated the graphs for clarity. In all, 96 h experiments for EREG, EGF, and NR are repeated for all the mentioned figures as controls.

We would like to highlight that the experiments for Figure 5 were conducted at a different time, in an independent set of experiments, and all 96 h experiments show results comparable to those observed in Figure 1.

Experiments for Figure S6 were also performed in parallel; thus, 96 h controls are repeated among scatter plots.

An explanatory statement has been added in the methods section.

Sentence 605-610
“Experiments for Figures 1, 2, 4, and 6 were performed in parallel; results are shown in separate figures and graphs for clarity. As experimental settings are compared to

96 h treatments with NR, EGF, or EREG, the corresponding control quantifications are repeated in the scatter plots. Experiments for Figure 5 were performed independently and at a separate time. Experiments for Figure S6 were also performed at the same time; thus, 96 h controls are repeated among the scatter plots"

6. The discussion appears not finalized. E.g. the paragraph discussing the clathrin-mediated endocytosis should be rewritten to clarify what is being said.

We have largely modified the manuscript in accordance with Life Science Alliance guidelines for submission and the reviewers' advice. There is now an improved, separate Discussion section.

7. A schematic picture of the findings and the author's interpretation on timing, dosing, and EGFR ligand-specificity to crypt/villus growth would be helpful.

We believe the main finding of this article is how the plasma membrane localization of EGFR is modified by ligands, regardless of the ligand used, triggering differential development of a proliferating crypt region vs a differentiated villus region. We designed schematic representations summarizing these findings in Figure 7.

8. There are spelling mistakes throughout the manuscript and parts of the materials and methods appear preliminary (e.g. "single organoid crypts (row 391), X-well). Was 50% Goat serum really used? Row 423 should be revised.

We have conducted an extensive grammar check on the current version of the manuscript; we apologize for any remaining mistakes.

We have rewritten the Goat Serum sentence; we actually dilute the Goat serum with IF buffer. This has been corrected.

Reviewer #3 (Comments to the Authors (Required)):

The manuscript submitted by the authors details their investigations into how EGFR ligands present in the media of in vitro grown small intestinal organoids affect their differentiation and proliferation. The manuscript definitely presents some necessary information related to how small intestinal organoids are grown and how the presence of specific types and amounts of EGFR ligands affect their growth and differentiation. However, the manuscript is difficult to understand in parts with many grammatical mistakes. Most concerning is the significant lack of figure references. There are a multitude of areas in the manuscript where conclusions and results are described but no reference to a specific figure (or reference to literature) is listed. This makes the manuscript difficult to understand and seeds a concerning level of doubt in the findings that are present. Detailed below are some of the major concerns I have with the manuscripts and what might be done to fix it. I strongly support this type of work as it is critical to have a good understanding of the media and ligands used to grow organoids if we are to use this tool as a means to better understand crypt growth. Therefore, I feel as though this manuscript could be considered for publication but would have to be heavily modified and then re-evaluated again before a decision can be made.

We thank the reviewer for their advice; the manuscript has been extensively revised in accordance with the Life Science Alliance guidelines and the reviewer's comments. Grammar has been revised, and figure/text references have been improved. We hope that these changes address the reviewer's concerns.

Major concerns:
EGF and EREG mediated endocytosis reduce EGFR PM localization and expression:

• The sentence on lines 145-147 states that most of the internalized EGF-647 is located in the crypt regains with few being observed in the villus regions. There is no figure reference to support this claim. The authors would need to provide data showing EGFR-647 internalization relative to CD44 vs AldoB immunofluorescence with quantification to say this.

Currently, in Figure S2A, we quantified EGF-647 internalization in both CD44- and AldoB-positive regions. This shows a clear enhancement of EGF-647 endocytosis in Crypts relative to villi. Please consider that, while they do not show CD44 or AldoB staining, Figure S2B and D and Figure 2B clearly show endocytosis in Ki-67+ regions, which also represent proliferative crypt regions.

• The result presented in line 148-150 is intriguing, however, the reason for the lack of EGF-647 internalization is likely due to saturating levels of EGF present in the media. This conclusion is hinted at later in the manuscript, but it would be good to put it here first.

We would like to clarify that EGF-647 is added to NR media at the end of the 96 h developmental window; thus, no other ligand is present in the media except the labeled EGF. This is stated in the methods section.

We have added this conclusion regarding Figure 1F (currently Figure 2A and B) as requested.

Sentence 176-181
“Absence of exogenous ligands likely allowed for accumulation of PM EGFR throughout the 96 h developmental window, while EREG medium promoted recycling to the plasma membrane, thereby maintaining high surface levels. As for organoids grown in EGF medium, it is likely that highly saturating EGF concentrations during 96 h were sufficient to completely deplete the cell surface of receptors, thus greatly reducing availability of PM EGFR for EGF-647 endocytosis”

• The sentence on line 173-176 is very confusing as it is written. The results clearly show decreased pEGFR in organoids grown in EGF or EREG, however, how CD44 fits into this is not adequately explained and does not due the data justice. It would be good to take a couple of sentences to describe the results a bit better and with more detail.

We apologize for this extremely unclear sentence. We have modified it accordingly.

CD44, in this case, serves as both a crypt marker and a plasma membrane marker that we use to normalize pEGFR staining. Traditionally, of course, an antibody against total, non-phosphorylated EGFR should be used for this normalization; however, no commercially available antibody we tested stained mSIOs.

Sentence 201-204
"Quantification of Immunofluorescence staining for pEGFR Y1068, normalized to CD44 membrane staining, showed increased phosphorylation in crypts grown without EGF or EREG, whereas organoids grown with either ligand showed reduced staining (Figure 2D)."

• The very last paragraph has some very important conclusions related to EGF secretion by Paneth cells and its effect on EGFR phosphorylation. However, as it is written, it is not clear how the bit related to media changes and pulses of EGF and EREG are relevant to the overall point of this section. This confusion is made worse by last sentence, which is especially confusing. As it is written, it doesn't fit with what the data shows.

We have written this paragraph again as follows:

Sentence 206-214
"EGFR endocytic cycle is fundamental to regulate receptor phosphorylation, allowing for degradation in late endosomes or interaction with protein tyrosine phosphatases (PTPs) within endomembrane compartments (Baumdick et al, 2015; Stanoev et al, 2018; Joshi et al, 2023). In turn, NR-grown organoids show an accumulation of pEGFR, likely due to endogenously expressed EGF, Paneth cells, or other ligands. Both EGF and EREG reduced total and surface expression levels of EGFR, and it is likely that the time frame between media changes allows for a recovery of EGFR at the PM, thus rendering developing organoids competent to sense ligands in the environment."

• It would be of use to also characterize what differentiated cell types are present in the various media conditions. This can be accomplished by immunofluorescence and would increase the support that the organoids are indeed differentiated. As an extension of this, it would also be good to examine markers of the intestinal stem cell population that should be present in the CD44+ crypt area of the organoids.

In the current version of the manuscript, we have expanded the cell populations examined by adding enteroendocrine cell staining across all our experimental conditions; these results are shown in Figure S6. We used antibodies against DCAMLK1 and CHGA to label two distinct enteroendocrine cell populations; however, we did not detect any changes across the conditions tested. It is important to note, as we have highlighted in the text, that enterocytes (AldoB+ cells) comprise about 70% of cells in the intestine. Our staining for CHGA and DCAMLK1 showed clear staining of individual positive cells; however, we could identify only 1 to 3 independent cells.

Stem cell markers for immunostaining are notoriously poor; we attempted staining for OLFM4 with mixed results, but overall we were unable to obtain proper staining or at least staining sufficient for quantification.

We also added the following statement in the discussion section:

Sentence 390-404
"In the present work, we attempt to bridge the vast knowledge currently available on the role of EGF-family ligands in intestinal development with the dynamics of EGFR signaling and the endocytic cycle (Koseska & Bastiaens, 2020; Jendrisek et al, 2026). To test this, we systematically added and removed EGF and EREG, quantified the amount of EGFR at the PM after treatments, and ultimately assessed the phenotypic effects on organoid development. CD44 and AldoB were used as markers for the proliferative crypt region and the differentiated villus region in mSIOs. We chose these markers not for their specificity regarding a certain subpopulation of differentiated cells within the intestinal epithelia, but for their widespread expression within cycling cells in the crypt for CD44 (eg. Lgr5+ stem cells and transit amplifying cells) (Wang et al, 2013) and for differentiated enterocytes for AldoB (Beumer et al, 2022; Cosovanu et al, 2022). For examining distinct cell populations, methods such as single-cell RNA sequencing or FACS sorting are better suited (Yang et al, 2025). However, these methodologies require a higher number of organoids, making it impractical to systematically test multiple conditions within an experimental setup"

Temporal modulation of EGF-family ligands during development alters villus differentiation.

• This section was weakest out of the whole manuscript. It was very difficult to even evaluate due to the significant lack of figure references. There were too many instances to exhaustively list here in which a result was described, but no figure was referenced. It can't be stressed enough that without a reference to a figure (even if it is repetitive) helps the reader stay on track with author's description of the results.

We apologize for the state of the previous manuscript version. We have corrected the missed reference and reworked much of this section. We also separated Figure 2 (Now Figure 3 and Figure 4) into two independent figures to make the conclusions in this section clearer. We have also added additional paragraphs discussing the results of this section (See below)

• A better description of how the organoids were split and when the media was added is also necessary here. Are these media conditions present right at splitting? Is the media change happening only one hour after the organoids are seeded? Or are the media changes occurring in organoids that are already established. A diagram detailing when the organoids are split and when the various media changes happen is necessary to be able to evaluate this section.

We realize that when describing experimental settings, we sometimes use the terms "single crypts" and "organoids" interchangeably. We have corrected this in the current manuscript.

All experiments start from the moment of seeding. Single crypts are obtained after mechanical disruption by pipetting and then seeded into a Matrigel droplet in a cell

culture well. Afterward, culture media is added. This sequence constitutes a “passage” and the starting point for all experiments in this work.

There are no additional passages over the next 96 h; only media changes at the stated times. As for the diagram, every vertical black line denotes a media change; often, above this black line is the timing at which this media change was performed after seeding. We have revised and enlarged the diagrams.

We have also added some details in the Methods addressing these concerns.

Sentence 497-504
“Media was changed every 48 h when single crypts were expanded in EGF-containing media for subsequent experiments. For treatments, NR medium was supplemented with 50 ng/mL of EREG (Biotechne, 1195-EP-025,) or 50 ng/mL NRG1 (Biotechne-377-HB-050/CF). For media changes, old media was aspirated and replaced with fresh media at established time points in each experiment (e.g., 1h, 24h, 48h). Organoids were briefly washed with warm AdDF+++ before adding fresh media if the media had a different composition from the original (e.g., 48h EGF media followed by 48h NR media).”

• The diagram present in Figure 2A indicates that EGF is added for the first hour and then it is removed and replaced with NR media. One hour doesn't seem a long enough period to result in any effect whatsoever on organoid growth.

We have added the following paragraph in the discussion:

Sentence 449-466
“Ligand removal and introduction experiments highlighted the robustness of EGFR-associated signaling, as ligand signaling inputs introduced at different stages of organoid development could trigger convergent phenotypic responses at the end of the 96 h developmental window. The more surprising result showed that 1 h of EGF incubation of seeded single crypts was sufficient to induce organoid development comparable to that induced by 96 h of constant EGF incubation. Experiments in cell culture monolayers often show that brief incubations with EGF, in the range of seconds to minutes, are sufficient to trigger downstream signaling activation as measured in Western Blots (Baumdick et al, 2015; Stanoev et al, 2018) or more sensitive methods such as FRET (Ponsioen et al, 2021; Weisheng et al, 2025). In contrast, how these “brief” signaling inputs ultimately affect biological systems in the long term (hours to days after signaling input) is not commonly examined, despite multiple examples in embryonic development (Sonnen & Janda, 2021). Based on our results, we can hypothesize that 1 h incubation with saturating EGF concentration was sufficient to initially deplete EGFR PM membrane levels and then allow for 95 h of EGFR replenishment. The presence of EGFR at the PM led to greater villus differentiation, resembling treatments with EREG or sub-saturating EGF concentrations.”

• I can't stress enough that there is likely good data in this section. However, without a proper explanation of when media changes are occurring in relation to seeding the organoids a proper evaluation of the experiments is not possible. This section would need to be rewritten, adding in a better description of the methods with a better

diagram and then re-evaluated before conclusions can be made related to the veracity of the results and any additional experiments needed.

As stated above, we have added additional text to the results and discussion sections, provided further clarity on how the treatments are performed in the methods section, separated the original figure, and clarified the distinction between seeding "organoids" and "single crypts". We hope this new manuscript addresses the reviewers concerns.

Sub-saturating concentrations of EGF enhance both crypt proliferation and villus differentiation:

• As seen in the last sections, this section lacked a few instances where a refecen to a figure is needed. For example, the sentence on line 282-283 clearly reference data but has no figure reference at the end. The same goes for lines 288-289.

We have corrected this: Figure 5B and Figure 5D, respectively.

• As stated above, a better characterization of crypt and villus differentiation beyond CD44 and AldoB is needed to evaluate how well the organoids are differentiating.

Enteroendocrine cells staining for this section are shown in Figure S6D; no significant changes were observed.

Multiple pulses of EGF deplete EGFR surface expression reducing villus differentiation:

• Line 306 to 308 lacks reference to a specific figure.

It has been corrected.

Minor concerns:

• In vitro should always be written in italics.

It has been corrected as requested.

• The references do not list all of the authors, but instead seem to only list a certain number of authors with no et al., after the last author. In the reference section, all authors associated with a given publication should be listed.

We have reformatted the whole manuscript, including references, in compliance with Life Science Alliance guidelines.

• There are a number of spots where conclusions are stated but no reference is listed. Below are two examples, but this is by no means an exhaustive list. Authors should always list a reference that was used to make a conclusion.

o The sentence ending on line 52 needs a reference so the reader can find the study that led the authors to reach this conclusion.

Reference has been added: Basak et al., 2017

o The same goes for the sentence ending on line 61.

Reference has been added: Sato et al., 2009

• The entire manuscript should be reviewed for grammatical mistakes; there were too many to provide an exhaustive list.

We made an extensive grammar revision; we hope the new manuscript addresses the reviewers' concerns.

• Abbreviations used in the manuscript should be spelt out first with the abbreviation in parentheses. All references after this can be made using the abbreviation.

We have corrected this as requested.

• The graphs in figure 2 should start with the data for 1 hour and then proceed up to 96.

We have changed the order of the shown treatments. Please bear in mind that all the experiments in the current figure 3, 4, and S5 are compared to the 96 h control treatment.

August 19, 2026

RE: Life Science Alliance Manuscript #LSA-2026-03733-TR

Dr. Mario O. Caracci
Max Planck Institute of Molecular Physiology
Systemic Cell Biology
Otto-Hahn-Straße 11
Dortmund, Nordrhein Westfalen 44227
Germany

Dear Dr. Caracci,

Thank you for submitting your revised manuscript entitled "Proliferation/Differentiation in intestinal organoids as a balance of ligand-modulated EGFR traffic". We returned this work to Reviewers 2 and 3 whose comments are below. As you will see, the reviewers are satisfied with a few minor requests. We would be happy to publish your paper in Life Science Alliance pending resolution of these requests and final revisions necessary to meet our formatting guidelines.

MANUSCRIPT ORGANIZATION AND FORMATTING:

To avoid unnecessary delays in the acceptance and publication of your paper, please read the following information carefully. Full guidelines are available on our Instructions for Authors page, https://www.life-science-alliance.org/authors

Submission of a paper that does not conform to Life Science Alliance guidelines will delay the acceptance of your manuscript.

-We suggest amending the manuscript title for clarity to: "Proliferation and differentiation in intestinal organoids are balanced by ligand-modulated EGFR trafficking"
-Please correct the abstract for grammar and consistency in tense: "basal phosphorylation" (Line 27) and "triggered" (Line 29). Kindly rephrase "(oncogenic) proliferation" to "proliferation and tumorigenesis" or similar. Finally, please include a concluding statement in the abstract by modifying the final sentence or adding a new one.
-Please add the X and Bluesky handles of your host institute/organization, as well as your own, and/or one of the authors, in our system.
-Please add the ORCID ID for the corresponding author--you should have received instructions on how to do so.
-Please remove the summary blurb from the manuscript file.
-Please add contact details for the corresponding author to the manuscript cover page.
-Figure/Supplementary Figure/Table legends should be placed at the end of manuscript file after the 'References' section.
-Figures 2-4: please make sure that panels in the figures correspond to the panels included in the figure captions.
-Please add a callout for Figures S6E to your main manuscript text.
-Because Figure S8 depicts uncropped blots, we invite you to upload these images as Source Data. If you prefer to retain this as a supplemental figure, please add a callout to this figure in the manuscript text other than the methods section.
-Please indicate the illumination source and acquisition software for microscopy, as well as image processing software (e.g. for masking) in the Materials and Methods section.

We encourage our authors to provide original source data, particularly uncropped/-processed electrophoretic blots and spreadsheets for the main figures of the manuscript. If you would like to add source data, we would welcome one PDF/Excel-file per figure for this information. These files will be linked as supplementary "Source Data" files.

It is Life Science Alliance policy that if requested, original data images must be made available to the editors. Failure to provide original images upon request will result in unavoidable delays in publication. Please ensure that you have access to all original data images prior to final submission.

If you are planning a press release on your work, please inform us immediately to allow informing our production team and scheduling a release date.

We welcome submissions of potential cover images for the issue of LSA in which your work would appear. If you have high quality images associated with this work, please feel free to email these, with a caption, to the journal office.

LSA encourages authors to provide a 30-60 second video where the study is briefly explained. These videos will be appear embedded with the manuscript online at Life Science Alliance, and on social media to promote the published paper and authors (for examples, see https://docs.google.com/document/d/1-UWCfbE4pGcDdcgzcmiuJl2XMBJnxKYeqRvLLrLSo8s/edit?

usp=sharing). Corresponding or first-authors are welcome to submit the video. Please submit only one video per manuscript. The video can be emailed to contact@life-science-alliance.org

FINAL FILES:

To upload the final version of your manuscript, please log in to your account: https://lsa.msubmit.net/cgi-bin/main.plex
You will be guided to complete the submission of your revised manuscript and to fill in all necessary information. Please get in touch in case you do not know or remember your login name.

The following items are required for acceptance.

-- An editable version of the final text (.DOC or .DOCX) is needed for copyediting (no PDFs).

-- High-resolution figure, supplementary figure and video files uploaded as individual files: See our detailed guidelines for preparing your production-ready images, https://www.life-science-alliance.org/authors

-- Summary blurb (enter in submission system): A short text summarizing in a single sentence the study (max. 200 characters including spaces). This text is used in conjunction with the titles of papers, hence should be informative and complementary to the title. It should describe the context and significance of the findings for a general readership; it should be written in the present tense and refer to the work in the third person. Author names should not be mentioned.

The license to publish form must be signed before your manuscript can be sent to production. A link to the license to publish form will be available to the corresponding author only. Please take a moment to check your funder requirements.

Reviews, decision letters, and point-by-point responses associated with peer-review at Life Science Alliance will be published, alongside the manuscript. If you do want to opt out of having the reviewer reports and your point-by-point responses displayed, please let us know immediately.

Thank you for your attention to these final processing requirements. Please revise and format the manuscript and upload materials as soon as you are able.

Thank you for this interesting contribution to the literature. We look forward to publishing your paper in Life Science Alliance.

Sincerely,

Tim Fessenden
Scientific Editor
Life Science Alliance

Reviewer #2 (Comments to the Authors (Required)):

The authors have addressed my comments well and the text has improved substantially. It remains a bit unclear why the changes in CD44+/AldoB+ areas in confocal imaging are not reflected by changes in the CD44/ALDOB ratios in qPCR or Western blot. An independent validation method of the crypt/villus representation measurement in the growth conditions, using any specific markers in an additional quantitative assay, would be beneficial to solidify this work in the future.

A minor points to be considered: The authors should state that "mRNA expression levels" (not only "expression levels" since that can also refer to protein expression) are addressed in Fig S1A-B and Fig S7.

Reviewer #3 (Comments to the Authors (Required)):

The manuscript submitted by the authors details their investigations into how EGFR ligands present in the media of in vitro grown small intestinal organoids affect their differentiation and proliferation. The manuscript in its current form is a vast improvement over the one initially submitted. Examining the effect growth factor and culture conditions have on organoid growth and is very valuable to the community. I feel that my initial concerns were adequately addressed. Therefore, I feel this manuscript would be suitable for publication provided the authors address two very minor issues.
• Line 228: The figure reference here should be Figure S3C, not S3B
• The authors show quantification of Paneth cells present in the organoids, but do not explain (unless I missed it) how the quantification was done. I saw in the methods that lysozyme was used. Did the authors quantify based on lysozyme IF signal? If so please add this to the manuscript and it would be beneficial to add the images as well.

August 19, 2026

RE: Life Science Alliance Manuscript #LSA-2026-03733-TR

Dr. Mario O. Caracci
Max Planck Institute of Molecular Physiology
Systemic Cell Biology
Otto-Hahn-Straße 11
Dortmund, Nordrhein Westfalen 44227
Germany

Dear Dr. Caracci,

Thank you for submitting your revised manuscript entitled "Proliferation/Differentiation in intestinal organoids as a balance of ligand-modulated EGFR traffic". We returned this work to Reviewers 2 and 3 whose comments are below. As you will see, the reviewers are satisfied with a few minor requests. We would be happy to publish your paper in Life Science Alliance pending resolution of these requests and final revisions necessary to meet our formatting guidelines.

MANUSCRIPT ORGANIZATION AND FORMATTING:

To avoid unnecessary delays in the acceptance and publication of your paper, please read the following information carefully. Full guidelines are available on our Instructions for Authors page, https://www.life-science-alliance.org/authors

Submission of a paper that does not conform to Life Science Alliance guidelines will delay the acceptance of your manuscript.

-We suggest amending the manuscript title for clarity to: "Proliferation and differentiation in intestinal organoids are balanced by ligand-modulated EGFR trafficking"

We gladly accept this suggestion.

We originally had a title like this, however LSA guidelines limit the number of characters it the title to 100. The suggested title has 107.

-Please correct the abstract for grammar and consistency in tense: "basal phosphorylation" (Line 27) and "triggered" (Line 29). Kindly rephrase "(oncogenic) proliferation" to "proliferation and tumorigenesis" or similar. Finally, please include a concluding statement in the abstract by modifying the final sentence or adding a new one.

Done.

-Please add the X and Bluesky handles of your host institute/organization, as well as your own, and/or one of the authors, in our system.

@mpimoph

-Please add the ORCID ID for the corresponding author--you should have received instructions on how to do so.

https://orcid.org/0009-0006-2772-8378

-Please remove the summary blurb from the manuscript file.

Done.

-Please add contact details for the corresponding author to the manuscript cover page.

Done.

-Figure/Supplementary Figure/Table legends should be placed at the end of manuscript file after the 'References' section.

Done.

-Figures 2-4: please make sure that panels in the figures correspond to the panels included in the figure captions.

Figure legends have been modified. We apologize for the mistake.

-Please add a callout for Figures S6E to your main manuscript text.

Done.

-Because Figure S8 depicts uncropped blots, we invite you to upload these images as Source Data. If you prefer to retain this as a supplemental figure, please add a callout to this figure in the manuscript text other than the methods section.

We have added references to this figure in the results section.

-Please indicate the illumination source and acquisition software for microscopy, as well as image processing software (e.g. for masking) in the Materials and Methods section.

Done.

We encourage our authors to provide original source data, particularly uncropped/-processed electrophoretic blots and spreadsheets for the main figures of the manuscript. If you would like to add source data, we would welcome one PDF/Excel-file per figure for this information. These files will be linked as supplementary "Source Data" files.

It is Life Science Alliance policy that if requested, original data images must be made available to the editors. Failure to provide original images upon request will result in unavoidable delays in publication. Please ensure that you have access to all original data images prior to final submission.

If you are planning a press release on your work, please inform us immediately to allow informing our production team and scheduling a release date.

We welcome submissions of potential cover images for the issue of LSA in which your work would appear. If you have high quality images associated with this work, please feel free to email these, with a caption, to the journal office.

LSA encourages authors to provide a 30-60 second video where the study is briefly explained. These videos will be appear embedded with the manuscript online at Life Science Alliance, and on social media to promote the published paper and authors (for examples, see https://docs.google.com/document/d/1-UWCfbE4pGcDdcgzcmiuJl2XMBJnxKYeqRvLLrLSo8s/edit?usp=sharing). Corresponding or first-authors are welcome to submit the video. Please submit only one video per manuscript. The video can be emailed to contact@life-science-alliance.org

FINAL FILES:

To upload the final version of your manuscript, please log in to your account: https://lsa.msubmit.net/cgi-bin/main.plex
You will be guided to complete the submission of your revised manuscript and to fill in all necessary information. Please get in touch in case you do not know or remember your login name.

The following items are required for acceptance.

-- An editable version of the final text (.DOC or .DOCX) is needed for copyediting (no PDFs).

-- High-resolution figure, supplementary figure and video files uploaded as individual files: See our detailed guidelines for preparing your production-ready images, https://www.life-science-alliance.org/authors

-- Summary blurb (enter in submission system): A short text summarizing in a single sentence the study (max. 200 characters including spaces). This text is used in conjunction with the titles of papers, hence should be informative and complementary to

the title. It should describe the context and significance of the findings for a general readership; it should be written in the present tense and refer to the work in the third person. Author names should not be mentioned.

The license to publish form must be signed before your manuscript can be sent to production. A link to the license to publish form will be available to the corresponding author only. Please take a moment to check your funder requirements.

Reviews, decision letters, and point-by-point responses associated with peer-review at Life Science Alliance will be published, alongside the manuscript. If you do want to opt out of having the reviewer reports and your point-by-point responses displayed, please let us know immediately.

Thank you for your attention to these final processing requirements. Please revise and format the manuscript and upload materials as soon as you are able.

Thank you for this interesting contribution to the literature. We look forward to publishing your paper in Life Science Alliance.

Sincerely,

Tim Fessenden
Scientific Editor
Life Science Alliance

Reviewer #2 (Comments to the Authors (Required)):

The authors have addressed my comments well and the text has improved substantially. It remains a bit unclear why the changes in CD44+/AldoB+ areas in confocal imaging are not reflected by changes in the CD44/ALDOB ratios in qPCR or Western blot. An independent validation method of the crypt/villus representation measurement in the growth conditions, using any specific markers in an additional quantitative assay, would be beneficial to solidify this work in the future.

Thank you for your kind comments. We believe that this discordance is intrinsic to the use of tissue like models, encompassing multiple cell types, expressing different markers at different levels or even subcellular distribution. FACS based methods or scRNA sequencing can of course provide a more comprehensive view of the cellular landscape within the organoids.

A minor points to be considered: The authors should state that "mRNA expression levels"

(not only "expression levels" since that can also refer to protein expression) are addressed in Fig S1A-B and Fig S7.

We have modified this as requested.

Reviewer #3 (Comments to the Authors (Required)):

The manuscript submitted by the authors details their investigations into how EGFR ligands present in the media of in vitro grown small intestinal organoids affect their differentiation and proliferation. The manuscript in its current form is a vast improvement over the one initially submitted. Examining the effect growth factor and culture conditions have on organoid growth and is very valuable to the community. I feel that my initial concerns were adequately addressed. Therefore, I feel this manuscript would be suitable for publication provided the authors address two very minor issues.

Thank you for your comments!

• Line 228: The figure reference here should be Figure S3C, not S3B

Done.

• The authors show quantification of Paneth cells present in the organoids, but do not explain (unless I missed it) how the quantification was done. I saw in the methods that lysozyme was used. Did the authors quantify based on lysozyme IF signal? If so please add this to the manuscript and it would be beneficial to add the images as well.

Yes, we have corrected this as requested. Single cells were identified from maximum intensity projections and counted, just as CHGA and DCAMKL cells. Representative images are already in Figure S1D for treatments showed in Figure 1.

August 21, 2026

RE: Life Science Alliance Manuscript #LSA-2026-03733-TRR

Dr. Mario O. Caracci
Max Planck Institute of Molecular Physiology
Systemic Cell Biology
Otto-Hahn-Straße 11
Dortmund, Nordrhein Westfalen 44227
Germany

Dear Dr. Caracci,

Thank you for submitting your Research Article entitled "Proliferation and differentiation in intestinal organoids are balanced by ligand-modulated EGFR trafficking". It is a pleasure to let you know that your manuscript is now accepted for publication in Life Science Alliance. Congratulations on this interesting work.

The final published version of your manuscript will be deposited by us to PubMed Central upon publication.

Your manuscript will now progress through copyediting and proofing. During proofing, kindly amend the Materials and Methods section to include information on illumination and acquisition/processing software for the digital lightsheet imaging.

We have identified that the primary corresponding author is from an institution that has elected to participate in an Open Access agreement with Life Science Alliance. Upon verification of the corresponding author's eligibility, all publication fees will be paid by their institution, and your article will be published immediately as open access under a CC-BY license.

Reviews, decision letters, and point-by-point responses associated with peer-review at Life Science Alliance will be published, alongside the manuscript. If you do want to opt out of having the reviewer reports and your point-by-point responses displayed, please let us know immediately.

IMPORTANT: If you will be unreachable at any time, please provide us with the email address of an alternate author. Failure to respond to routine queries may lead to unavoidable delays in publication.

Scheduling details will be available from our production department. You will receive proofs shortly before the publication date. Only essential corrections can be made at the proof stage so if there are any minor final changes you wish to make to the manuscript, please let the journal office know now.

DISTRIBUTION OF MATERIALS:
Authors are required to distribute freely any materials used in experiments published in Life Science Alliance. Authors are encouraged to deposit materials used in their studies to the appropriate repositories for distribution to researchers.

You can contact the journal office with any questions, contact@life-science-alliance.org

Again, congratulations on a very nice paper. I hope you found the review process to be constructive and are pleased with how the manuscript was handled editorially. We look forward to future exciting submissions from your lab.

Sincerely,

Tim Fessenden
Scientific Editor
Life Science Alliance
